# Bilberry (*Vaccinium myrtillus* L.) Extracts Comparative Analysis Regarding Their Phytonutrient Profiles, Antioxidant Capacity along with the In Vivo Rescue Effects Tested on a *Drosophila melanogaster* High-Sugar Diet Model

**DOI:** 10.3390/antiox9111067

**Published:** 2020-10-30

**Authors:** Andreea-Adriana Neamtu, Rita Szoke-Kovacs, Emoke Mihok, Cecilia Georgescu, Violeta Turcus, Neli Kinga Olah, Adina Frum, Ovidiu Tita, Carmen Neamtu, Zsombor Szoke-Kovacs, Zoltan Cziaky, Endre Mathe

**Affiliations:** 1 Doctoral School of Biomedical Sciences, University of Oradea, 410087 Oradea, Romania; aneamtu94@gmail.com; 2 Doctoral School of Molecular Cell Biology and Immunology, Faculty of Medicine, University of Debrecen, H-4032 Debrecen, Hungary; takacsrita@unideb.hu; 3 Doctoral School of Animal Science, Faculty of Agricultural and Food Sciences and Environmental Management, University of Debrecen, H-4032 Debrecen, Hungary; mihokemoke@uni.sapientia.ro; 4 Faculty of Agricultural Science, Food Industry and Environmental Protection, “Lucian Blaga” University of Sibiu, 550012 Sibiu, Romania; adina.frum@ulbsibiu.ro (A.F.); ovidiu.tita@ulbsibiu.ro (O.T.); 5 Faculty of Medicine, “Vasile Goldis” Western University of Arad, 310045 Arad, Romania; violeta.turcus@uvvg.ro (V.T.); neamtu.carmen@uvvg.ro (C.N.); 6 Faculty of Pharmacy, “Vasile Goldis” Western University of Arad, 310045 Arad, Romania; olah.neli@uvvg.ro; 7 Doctoral School of Nutrition, Faculty of Agricultural and Food Sciences and Environmental Management, University of Debrecen, H-4032 Debrecen, Hungary; szoke.zsombor@agr.unideb.hu; 8 Agricultural and Molecular Research and Service Institute, University of Nyiregyhaza, H-4400 Nyíregyháza, Hungary; cziaky.zoltan@nye.hu; 9 Faculty of Agricultural and Food Sciences and Environmental Management, University of Debrecen, H-4032 Debrecen, Hungary

**Keywords:** bilberry, phytochemical, antioxidant activity, flavonoid, polyphenol, hemolymph trehalose, insulin resistance, metabolic syndrome, *Drosophila melanogaster*

## Abstract

Bilberries (*Vaccinium myrtillus* L.) have been reported to hold a plentitude of health-promoting properties beyond basic nutrition, mainly attributed to their anthocyanin content and antioxidant activity. In this article, we built the phytochemical profile of three wild bilberry fruit extract formulations (aqueous, methanolic, and hydro-methanolic) using UHPLC-ESI-MS/MS putative analysis, identifying 88 individual phytochemicals, mainly flavonoids (total content 8.41 ± 0.11 mg QE/g dw), free amino acids, polyphenols (total content 21.68 ± 0.19 mg GAE/g dw), carboxylic acids, and vitamins. Furthermore, the antioxidant activity of the extract was assessed, reaching 78.03 ± 0.16% DPPH free radical scavenging activity, comparable to literature values determined for bilberry extracts of other origin. Due to the increased prevalence of metabolic syndrome and based on the reviewed benefits of bilberries, we tested the most potent formulation of our bilberry extracts in this biological context. The in vivo rescue effect of a bilberry extract supplemented diet on *Drosophila melanogaster* was assessed by monitoring biochemical and genomic markers. Hemolymph trehalose levels were halved upon addition of 3% hydro-methanolic bilberry extract to a high-sugar (1.5 M sucrose) diet, as compared to the non-supplemented high-sugar diet. Noteworthy, the rescue seen for flies kept on the bilberry extract supplemented high-sugar diet appeared to parallel the trehalose levels observed in the case of the control diet (50 mM sucrose) flies. Moreover, next to the trehalose-lowering type of in vivo effects, other gene expression related rescues were also detected for genes such as *InR*, *Akh*, *AstA*, *AstC*, *Irk*, *Npc2g*, and *CCHa2* upon supplementation of the high-sugar diet with our hydro-methanolic bilberry fruit extract. Our findings suggest that such a bilberry fruit extract could generate physiological and genomic type of compensatory mechanisms so that further translational approaches would advance the understanding of some human specific pathological conditions.

## 1. Introduction

Bilberries (*Vaccinium myrtillus* L.) are the native European blueberries, closely related to the North American blueberry species (*Vaccinium corymbosum* L.), both part of the widespread genus *Vaccinium* containing over 200 species [1]. Berries constitute a large group of functional foods, also nowadays known as “superfoods”, whose consumption delivers several health benefits beyond basic nutrition, mainly attributed to their anthocyanin content holding mainly antioxidant but also recently characterized antiglycoxidant properties [2,3]. Despite their similarities, berries differ in their phytochemical content, such as flavonoids and anthocyanins, of both plants and fruit [4,5,6]. The quantity of the main anthocyanins found in bilberries is more than twice the one found in blueberries [7]. Furthermore, anthocyanin content seems to be higher in wild bilberries as compared to the cultivated ones [8,9,10].

The biological effects of individual anthocyanins have been studied in detail; however, that does not hold true for the whole bilberry fruit extract. Complex interactions of multiple components within the food matrix may differ significantly in outcome when compared to a single purified constituent or constituents [11]. Hence, there is a need to evaluate the observed biological properties as a result of not only additive but also complementary synergistic and/or antagonistic effects, or any combination thereof, of all phytochemicals present [11]. In line with this rationale, we decided to focus our study on the whole bilberry fruit extract.

Bilberries have been observed to hold a plentitude of health-promoting properties. Research studies support their beneficial effects, among which antioxidant, anti-obesity, anticarcinogenic, cardioprotective, anti-inflammatory, hypoglycemic, antimicrobial, and vision improvement [12,13,14].

Based on the health-promoting properties of bilberries, we decided to study the extract in a *Drosophila melanogaster* model for insulin resistance and type II diabetes [15]. The first paper that explicitly modelled type II diabetes in *Drosophila* tested the role of a high-sugar diet on the acquisition of insulin resistance [16]. Characteristics of insulin resistance were assessed as rearing flies on high-sugar diet resulted in the overexpression of *dilp 2*,*3*,*5* RNA and circulating DILP2. Despite increased levels of DILP(s) in circulation, sugar levels remained elevated—a feature resembling mammalian insulin resistance. Moreover, decreased levels of phospho-Akt were observed in response to administration of exogenous insulin in adult flies reared on a high-sugar diet, suggesting a decreased capacity of response to insulin signalling following chronic uptake of dietary sugar. Finally, flies exposed to a high-sugar diet exhibited elevation of free fatty acid and triglyceride levels as compared to the flies raised on a control isocaloric diet, and the morphology of lipid storage changed, to exhibit fewer, larger droplets [17]. Together, these studies showed that flies kept on a chronic high-sugar diet are hyperglycemic, insulin-resistant, and obese, constituting a relevant model to assess our bilberry fruit extract health promoting properties [18,19].

This is highly relevant nowadays, as in recent years, people consume increasingly higher amounts of carbohydrates, to reach what Bovi et al. suggestively called “the pandemic of sucrose intake” [20]. Added sugars represent empty calories, as they only supply food energy but no other nutrients. They increase the risk of developing obesity, inflammation, cardiovascular disease, hypertension, diabetes, insulin resistance, obesity-related cancers, and metabolic syndrome, a non-communicable disease responsible for morbidity and mortality predominantly in the developed world [21]. It is defined by the World Health Organization as a pathology characterized by abdominal obesity, insulin resistance, hypertension, and hyperlipidemia [22]. The incidence of metabolic syndrome in the United States is about one in three people, reaching epidemic proportions [23].

With growing attention dedicated to harvesting and consuming local foods, we find it important to build the chemical profile, including chemo-mapping, antioxidant activity, and polyphenol and flavonoid content of bilberry fruit growing in the Carpathian Mountains, in the central part of Romania, which is a novel analysis and never before made the subject of a scientific article to the best of our knowledge. We also examine the rescue effects generated by our bilberry fruit extract in relation to the modifications caused by a high-sugar diet, using *Drosophila melanogaster* as a nutritional genetic model.

## 2. Materials and Methods

### 2.1. Chemicals and Reagents

Gallic acid, quercetin, formic acid, tryptophan, catechin, epigallocatechin, chlorogenic acid, vanillin, epicatechin, cyanidin-3-*O*-glucoside, 4-coumaric acid, isoquercitrin, myricetin, quercitrin, and naringenin, Folin-Ciocalteu reagent, DPPH (2,2-diphenyl-1-picrylhydrazyl), methanol analytical grade, aluminum chloride, sodium acetate, and sodium carbonate were purchased from Sigma Aldrich (Merck; Darmstadt, Germany).

### 2.2. Extracts Preparation

Extracts of bilberry (*Vaccinium myrtillus* L.) were prepared in three formulations from ripe fruit of wild bilberry collected in three consecutive years from the same alpine ecosystem in Făgăraș (South Carpathian) Mountains, Sibiu county, Romania, in July 2017, 2018, and 2019. The further described extracts were prepared from a mixture of samples of all three harvests, frozen and stored at −20 °C, then oven dried at 40 °C, until a constant mass was achieved, grinded on a domestic mill, and pooled together in equal quantities.

In the preparation of the extracts, the solvent varied as follows: E1—distilled water (aqueous extract); E2—methanol (methanolic extract); E3—distilled water:methanol mixture in 1:1 ratio (hydro-methanolic extract). For each extract, 500 mg of fruit powder was extracted using 10 mL of the specific solvent, in the ultrasound bath for 30 min, at 40 °C. Then, vacuum filtration was performed through Grade 1 (11 µm pore size) Whatman filter paper and brought to scale in 10 mL volumetric flasks.

Post-preparation, the extracts were stored in the fridge at 4 °C, in tinted glass bottles. Prior to experimental usage, the extracts were allowed to reach room temperature and shaken thoroughly.

### 2.3. Phytonutrient Profile Determination Via UHPLC-ESI-MS/MS Analysis

A Dionex Ultimate 3000RS ultrahigh pressure liquid chromatography (UHPLC) system equipped with a Thermo Scientific Accucore C18 column, L./I.D. of 100/2.1 mm, and a particle size of 2.6 μm, was coupled to a Thermo Q Exactive Orbitrap mass spectrometer (MS) using an electrospray ionization source (ESI), and the measurement accuracy was within 5 ppm. The mass spectrometer was operated at 320 °C capillary temperature, 4.0 kV in positive mode and 3.8 kV in negative mode of spray voltage, and a resolution of 35,000 in the case of MS, while it was 17,500 for MS/MS. The 100–1500 *m*/*z* was the scanned mass interval. For MS/MS scans, the collision energy was 35 NCE. The difference between measured and calculated molecular ion masses was always below 5 ppm.

For both the positive and negative ionization modes of UHPLC separation, an eluent A (1000 mL of water containing and 1 mL of formic acid) and eluent B (1000 mL of methanol and 1 mL of formic acid) combination was used.

The flow rate was set to 200 μL/min, and the same gradient elution program was used both positive and negative ionization mode type of determinations (0–3 min, 95% A, 3–43 min, 95→0% A; 43–61 min, 0% A; 61–62 min, 0→95% A; 62–70 min, 95% A). An amount of 5 μL of aqueous or hydro-methanolic ripe bilberry fruit extracts was injected at every run.

In the analysis, the following isolated compounds were used as standards: gallic acid (3,4,5-trihydroxybenzoic acid), tryptophan, catechin, epigallocatechin, chlorogenic acid (3-*O-*caffeoylquinic acid), vanillin, epicatechin, cyanidin-3-*O-*glucoside (kuromanin, asterin, chrysanthemin), 4-coumaric acid, isoquercitrin (hirsutrin, quercetin-3-*O-*glucoside), myricetin, quercitrin (quercetin-3-*O-*rhamnoside), and naringenin.

The data were acquired and processed using Thermo Trace Finder 2.1 software (Thermo Fisher Scientific Inc.; Waltham, MA, USA) based on own and internet databases: Metlin (La Jolla, CA, USA), Mass Bank of North America (Davis, CA, USA), *m*/*z* Cloud (HighChem; Bratislava, Slovakia). After processing, the results were manually checked using Thermo Xcalibur 4.0 software (Thermo Fisher Scientific Inc.; Waltham, MA, USA). The phytochemicals found in the extracts were identified on the basis of our previous published works or data found in literature using the exact molecular mass, isotopic pattern, characteristic fragmentation ions, and retention time.

### 2.4. Spectrophotometric Determication of the Total Polyphenol and Flavonoid Content, and the Antioxidant Activity of the Extracts

#### 2.4.1. Total Polyphenol Content Determination

The total polyphenols content was determined by the Folin–Ciocalteu method [24]. An amount of 0.4 mL of extract was mixed with 1 mL Folin–Ciocalteu reagent, 15 mL distilled water, and 2 mL of sodium carbonate solution (290 g/L). The mixture was shaken and then kept in a water bath at 40 °C for 20 min. The absorbance was measured at 760 nm using a Cecil CE 1021 UV-Vis spectrophotometer. The calibration curve was determined, showing a linear range for concentrations of 0.9–4.3 µg/mL gallic acid. The total polyphenol content of extracts was expressed as mg gallic acid equivalents (GAE)/g dry weight (dw), using the following equation based on the calibration curve: y = 73.773x − 0.0198 (R^2^ = 0.9937), where x was the absorbance recorded at 760 nm and y was the concentration expressed as mg GAE/g dw.

#### 2.4.2. Total Flavonoid Content

The total flavonoid content was determined by using a colorimetric method adapted after the Romanian Pharmacopoea [25]. In a 25 mL volumetric flask, 1 mL of the extract was mixed with 5 mL of a sodium acetate (100 g/L) and 3 mL of an aluminum chloride (25 g/L) solution and methanol to 25 mL. The mixture was shaken and left to stand for 15 min at room temperature before the analysis. The absorbance was recorded at 430 nm, using a Cecil CE 1021 UV-Vis spectrophotometer, and the results were expressed as mg quercetin equivalent (QE)/g dw. The calibration curve was determined, showing a linear range for concentrations 8.0–40.2 µg/mL quercetin. The equation based on the calibration curve: y = 27.627x − 0.0374 (R^2^ = 0.993), where x was the absorbance and y - the concentration expressed as mg QE/g dw.

#### 2.4.3. Antioxidant Activity through DPPH Free Radical Scavenging Assay

The free radical 2,2-diphenyl-1-picrilhydrazyl (DPPH) was used to test the ability of our extracts to act as free radical scavengers or hydrogen donors and, hence, the evaluation of their antioxidant activity. DPPH free radical scavenging activity was determined by using the method proposed by Tylkowski and colleagues [26]. A stock solution of 25 µg/mL DPPH in methanol was prepared and kept at 4 °C in the dark for 2 h before usage. An amount of 970 µL of DPPH stock solution was added onto 30 µL of extract. The absorbance was recorded at 517 nm, using a Cecil CE 1021 UV-Vis spectrophotometer, and the results were expressed as percentages. The calibration curve was linear for the range of DPPH concentrations of 0.25–250 µg/mL. The equation based on the calibration curve: y = 0.0047x + 0.013 (R^2^ = 0.996), where x was the absorbance and y—the concentration expressed as µg DPPH per mL. The DPPH radical scavenging activity was determined by using the following formula: RSA (%) = [(C_0_ − C_1_)/C_0_]·100, where: RSA represents the DPPH radical scavenging activity (%), C_0_ represents the concentration of the DPPH stock solution (µg/mL), and C_1_ represents the DPPH concentration in the sample (µg/mL).

### 2.5. Drosophila Melanogaster Strains and Feeding Design

Wild type *Drosophila melanogaster* Canton Special flies were used in all experiments. The flies were acquired from Bloomington Drosophila Stock Center (Indiana University, USA). Flies were reared on a standard medium containing 6% (*w/v*) yeasts, 4% (*v/v*) molasses, 1.25% (*w/v*) agar, and 0.4% (*v/v*) propionic acid (as a mold growth inhibitor). For larvae cultivation, a bottle of flies (10–15 males + 10–15 females) was placed at 25 °C. The flies were allowed to lay eggs for 2–4 h, then the bottle was cleared of flies and incubated for 3–4 days at 25 °C, or until third instar larvae were visible. Some water was then added to the bottle with larvae and let sit for 10 min. The 3rd instar larvae crawled to the walls, were collected using a brush, and placed into a petri dish containing PBS. Then, larvae were gently washed and transferred to a mesh basket and placed tissue paper to dry. About 200 3rd instar larvae were transferred to Holidic medium, containing 50 mM sucrose, and prepared as described by Piper and colleagues [27]. After eclosion, the flies were transferred to experimental media, namely: Holidic media (control); Holidic media with additional 1.45 M sucrose (high-sugar diet); Holidic media and 3% (*v/v*) aqueous bilberry extract E1 (control + bilberries); Holidic media and additional 1.45 M sucrose and 3% (*v/v*) aqueous bilberry extract E1 (high-sugar diet + bilberries). The files were reared on such experimental media for 12 days at 25 °C, and further analyzed as described in the forthcoming paragraphs.

### 2.6. Drosophila Melanogaster Two-Choice Feeding Preference Assay

Flies were first starved for a day and then provided with the choice of two foods, presented on a Petri dish at concentrations that were used in our experiments. The two food choices also contained different tasteless colored substrates (sulforhodamine B dye—red and erioglaucine dye—blue). The feeding was carried out in darkness (to exclude any influence of color preference), and the abdomen of the flies was inspected visually on the next day. The flies feeding exclusively on one of the substrates were featuring the corresponding red or blue abdomens, while the flies feeding on both substrates presented purple coloured abdomens. Counting the flies can be used to determine the feeding preference index: PI (red) = [N(red) + 0.5N(purple)]/[N(red) + N(blue) + N(purple)].

### 2.7. Drosophila Melanogaster Hemolymph Glycemia Measurement

To determine the hemolymph specific trehalose concentration, 20 flies (10 females and 10 males) per sample were briefly knocked down with CO_2_, and then their thorax was poked under the wing or next to the legs. The fly bodies were then transferred to a haemolymph collection tube placed on ice and centrifuged for 6 min at 3000× *g* at 4 °C. A total of 0.5 μL of hemolymph was mixed with 100 μL of the Glucose (HK) Assay Kit (Sigma; G3293–50ML). A total of 0.5 μL of Porcine Trehalase (Sigma, T8778) per 10 μL of reaction was added to the mixture, incubated at 37 °C for 18 h, and analyzed with a fluorescent plate reader measuring absorption at 340 nm wavelength. Standard curves were generated from D-glucose (0–1000 mg/mL) standards for each trial.

### 2.8. Analysis of mRNA Levels of Neurohormonal Drosophila Melanogaster Genes by qRT-PCR

#### 2.8.1. Total RNA Extraction and cDNA Synthesis

Male flies were decapitated after immersion in liquid nitrogen. We prepared three total RNA samples per treatment. For each sample 50 fly heads were placed into RNA stabilization solution (TRIzol) and kept at 4 °C until RNA extraction. The heads were homogenised in 1.5 mL RNase-free Eppendorf tubes containing 1 mL TRIzol reagent using a 1.5 mL pestle (Astral, RNase- and DNase-free; Thermo Fisher Scientific Inc.; Waltham, MA, USA). Samples were incubated for 15 min at room temperature and centrifuged for 10 min at 12,000× *g* at 4 °C. An amount of 800 µL of supernatant was decanted off and 200 µL of chloroform added. Tubes were shaken vigorously for 15 s, incubated at room temperature for 3 min and centrifuged for 20 min at 12,000× *g* at 4 °C. The aqueous phase was transferred to a Direct-zol™ RNA MiniPrep column (Zymo Research R2070; Freiburg, Germany), and all other steps were performed according to the manufacturer’s protocol. Extraction was followed by a DNase treatment (DNaseI, Invitrogen; Thermo Fisher Scientific Inc.; Waltham, MA, USA) to eliminate potential genomic DNA in the sample parameters. RNA was then stored at −80 °C before further processing. The quality and quantity of RNA were assessed by Qubit RNA HS Assay (Thermo Fisher Scientific Inc.; Waltham, MA, USA) and with an Agilent Bioanalyser RNA 6000 Pico Kit (Agilent Technologies, Inc.; Santa Clara, CA, USA). Synthesis of cDNA and subsequent quantification of mRNA levels for specific genes were performed using SuperScript™ III First-Strand Synthesis SuperMix for qRT-PCR (Thermo Scientific 11752050; Waltham, MA, USA). The cDNA samples were stored at −20 °C until further use.

#### 2.8.2. Primer Design

The real-time qPCR assays were designed by using the Roche Universal Probe Library (Roche Molecular Systems, Inc.; Basel, Switzerland). Primers used for the analysis were designed by the Roche Probe Finder Assay Design Software [28]. The designed primers covered an exon–intron boundary to prevent genomic DNA amplification. The PCR primer sequences are shown in Table S7.

#### 2.8.3. Reverse Transcription qPCR

On a Roche Light Cycler 480 Real Time PCR System, triplicate first strand cDNA aliquots for each sample served as templates for RT-qPCR using the Light Cycler 480 Probes Master PCR Master Mix (Roche Molecular Systems, Inc.; Basel, Switzerland). The amplification reactions were performed in 10 mL total volumes with 3 mL of cDNA (diluted 1:100) and 100 nM of each primer, in 384-well optical plates (LightCycler^®^ 480 Multiwell Plate 384, Roche Molecular Systems, Inc.; Basel, Switzerland). The program conditions were: pre-incubation 1 cycle (95 °C, no acquisition, 10 min, 4.8 ramp rate), amplification 45 cycles (95 °C, no acquisition, 10 s, 4.8 ramp rate; 60 °C, single acquisition, 30 s, 2.5 ramp rate; 72 °C, no acquisition, 1 s, 4.8 ramp rate), and cooling 1 cycle (40 °C, no acquisition, 30 s, 2.5 ramp rate). Reverse transcription-qPCR efficiency was determined for each gene with the slope of a linear regression model. Relative standard curves for the gene transcripts were generated with serial dilutions of cDNA.

#### 2.8.4. Data Mining and Selection of Reference Gene Candidates

Expression levels were determined as the number of cycles needed for the amplification to reach a fixed threshold in the exponential phase of the PCR reaction [29]. The number of cycles is referred to as the quantification cycle (Cq) value, the standard name for the threshold cycle or crossing point value according to the RDML guidelines [30]. The threshold was set at 0.045 for all genes, and the corresponding Cq values were transformed into quantities via the standard curve using PCR efficiencies according to [31].

## 3. Results

### 3.1. Chemical Analysis of the Extracts

**Figure 1:**
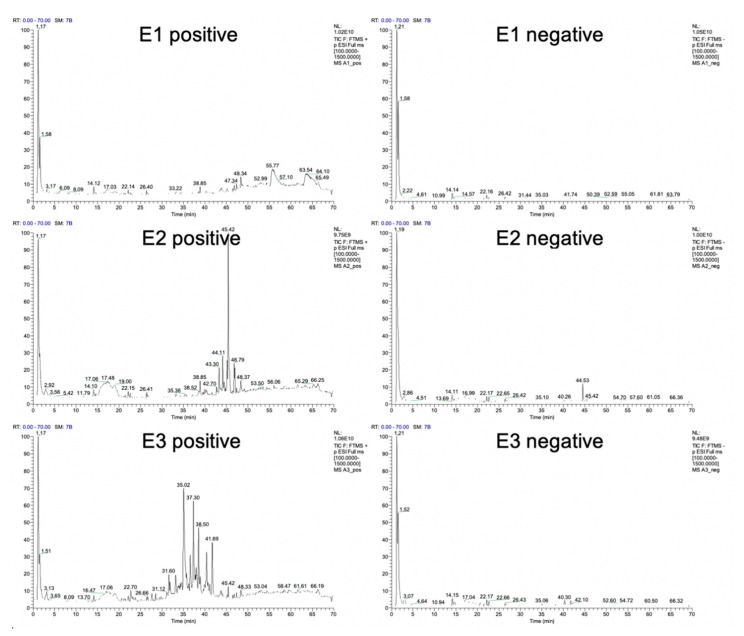
Ion chromatograms in positive and negative mode obtained using UHPLC-ESI-MS/MS.

The aqueous (E1), methanolic (E2), and hydro-methanolic with 1:1 water:methanol ratio (E3) extracts of bilberry ripe fruit underwent UHPLC-ESI-MS/MS qualitative analysis in order to determine their phytonutrient profile. The chromatograms in positive and negative ionization mode are shown in Figure 1 for all analyzed extracts, with labeled retention times of major peaks. The data recorded can be found in Table S1 (E1), Table S2 (E2), and Table S3 (E3). The fragmentation patterns used in the putative identification of the total of 88 individual phytochemicals are summarized in Table S4.

This analysis revealed several differences in the composition of the extracts of the bilberry ripe fruit depending on the applied extraction protocol used. The comparative analysis of the composition is presented in Table 1*,* and it also contains the chemical classification of the compounds. A total of 88 biomolecules were identified in the ripe bilberry fruit through our analysis. Out of these, the most phytochemicals, 81 different compounds, were identified in the hydro-methanolic extract, while 79 compounds were identified in the methanolic, and the lowest number, namely 65 compounds, were observed in the aqueous extract.

**Figure 2:**
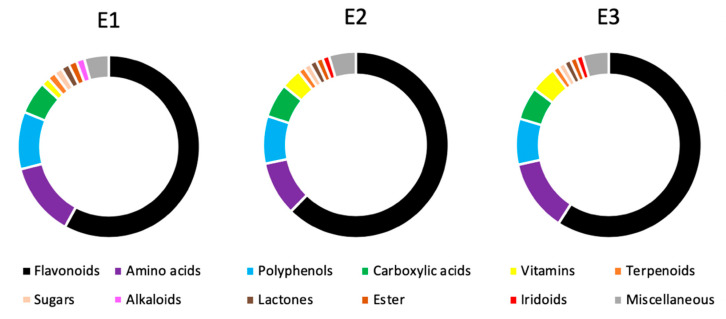
Composition based on the number of representatives of each class of compounds in the bilberry ripe fruit aqueous (**E1**), methanolic (**E2**), and hydro-methanolic (**E3**) extracts.

The chemo-mapping of the extracts based on the number of members of each chemical class is also summarized in Figure 2. More than half of the phytochemicals identified in the extracts are representatives of the flavonoid class—48 compounds out of 88. The classes of amino acids (12 compounds), polyphenols (6 compounds), carboxylic acids (5 compounds), and vitamins (4 compounds) were also revealed through the qualitative analysis of the composition of bilberry ripe fruit extracts.

Looking at the chemical classification and the identified number of the bilberry specific phytoconstituents, it seems obvious that the flavonoids represent the largest category followed by amino acids, polyphenols, carboxylic acids, and vitamins (Table 1, Figure 2).

It is also interesting that choline, the uniquely identified alkaloid in our study, was present only in the aqueous extract (E1), while the 7-deoxyloganic acid representing the iridoids was observed exclusively in the methanolic (E2) and the hydro-methanolic (E3) extracts. Other differences regarding the composition of the phytonutrient profiles of the assessed bilberry extracts were also evident (Table 1), confirming the importance of the applied extraction conditions.

To continue the phytonutrient profile characterization we have shown that from the three extraction formulations, the hydro-methanolic extract (E3) presented the highest amounts of total polyphenols (21.68 mg GAE/g dw) and flavonoids (8.41 mg QE/g dw), closely followed by the methanolic extract (E2), while the lowest quantities of these compounds were found in the aqueous extract (E1), at roughly half of the content. Thus, the hydro-alcoholic solvent was proven to extract the compounds of interest more efficiently than methanol or water alone (Table 2).

The in vitro antioxidant activity of the extracts was determined based on the DPPH free radical scavenging assay. The obtained antioxidant activity data suggest a trend that parallels the total polyphenolic and flavonoid content of the analyzed extracts (Table 2), meaning that the hydro-methanolic extract (E3) having the highest total polyphenol and flavonoid contents showed the strongest antioxidant activity, while the aqueous extract (E1) specific lowest total polyphenol and flavonoid content was associated with the most reduced antioxidant activity.

### 3.2. In Vivo Drosphila Melanogaster Studies of the Rescue-Effect of the Hydro-Methanolic Extract (E3)

Seeing the plenitude of phytonutrients associated with our bilberry extracts, and preparing to analyze the associated biological effects, we set to avoid the false positive data due to the preference or avoidance of the experimental fruit fly culture media. Therefore, a two-choice preference assay was carried out, and the feeding preference index was determined for each culture media. There was no preference or avoidance observed towards any of the experimental media used in further experiments, with an average preference index of about 0.5 (Table S9).

**Figure 3:**
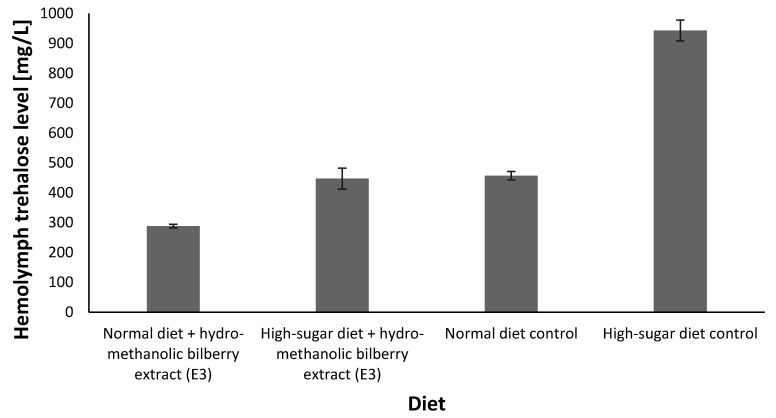
Hemolymph trehalose levels for Drosophila melanogaster Canton Special exposed to high-sugar diet with and without supplementation of hydro-methanolic bilberry extract (E3). Data are reported as average of triplicate experiments, based on Table S8.

Experiemntal data concerning the individual phytonutrients present in biberry have been shown to generate a strong antidiabetic physiological effect (Table S6). Our data support this finding, so that flies reared on a high-sugar diet and supplemented with hydro-methanolic bilberry extract (E3) showed a reduction in hemolymph trehalose, reaching similar levels to those observed in the normal diet control (Figure 3), and significantly lower levels than those of flies reared on the high-sugar diet without extract supplementation. The choice for the hydro-methanolic bilberry ripe fruit extract (E3) formulation was based on its highest polyphenol and flavonoid content, highest antioxidant activity (Table 2), and highest number of phytochemicals present (Table 1).

**Figure 4:**
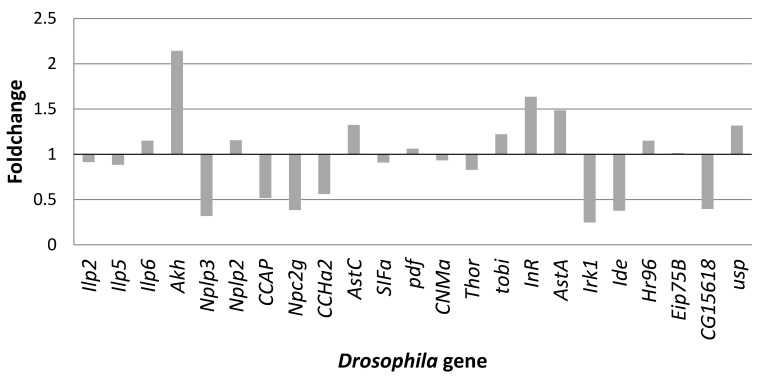
Foldchange in neuropeptide gene expression of wild-type *Drosophila melanogaster* reared for 12 days on a high-sugar diet, as compared to a normal diet control. Data reported as average of triplicate experiments, as ratio of gene expression values on high-sugar diet to gene expression levels on normal diet control.

Additional to the doubled hemolymph trehalose levels exhibited by flies reared on the high-sugar diet as compared to the normal diet control, such flies also featured modified gene expression profiles for a set of genes encoding for neuropeptides (Figure 4). Among the analyzed genes, 11 exhibited elevated mRNA levels, with the highest increase observed in the case of *Akh* (above 2-fold) followed by *InR* and *AstA* (about 1.5-fold). Genes such as *Ilp6*, *Nplp2*, *AstC*, *tobi*, *Hr96*, and *usp* exhibited foldchanges between 1.14 and 1.35, while *pdf* and *Eip75B* showed very slight overexpression, with values close to their constitutive levels. From the analyzed genes, pronounced downregulation of mRNA expression was observed in the case of *Nplp3*, *CCAP*, *Npc2g*, *CCHa2*, *Irk1*, *Ide*, and *CG15618*, with less than half of the gene expression levels when the flies were reared for 12 days on a high-sugar diet as compared to the normal diet control. Slightly downregulated expression levels (below 20% decrease) were also apparent for *Ilp2*, *Ilp5*, *SIFa*, *CNMa*, and *Thor* genes.

**Figure 5:**
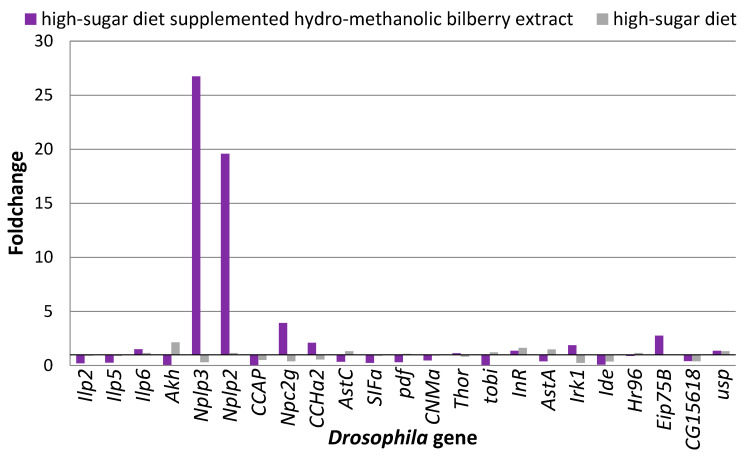
Foldchange in neuropeptide gene expression of wild-type *Drosophila melanogaster* reared for 12 days on a high-sugar diet, supplemented or not with 3% hydro-methanolic extract (E3). Data reported as average of triplicate experiments, as ratio of gene expression values on high-sugar diet to gene expression levels on normal diet control.

Upon high-sugar diet supplementation with 3% hydro-methanolic bilberry extract (E3), the foldchanges in mRNA expression of the analyzed neuropeptide-encoding genes were more dramatic (Figure 5). The *Nplp2* and *Nplp3* genes were more than 20-fold overexpressed, while for genes such as *Npc2g*, *CCHa2*, and *Eip75B*, the overexpression exceeded a 2-fold increment as compared to their corresponding controls. Moreover, we could observe the severe downregulation of other genes like *Ilp2*, *Ilp5*, *Akh*, *CCAP*, *SIFa*, *tobi*, and *Ide*, whose mRNA expression decreased below 30% of the respective control.

## 4. Discussion

The chemical evaluation of the three bilberry extracts revealed the hydro-methanolic extract (E3) containing the highest number of phytochemicals, and showing the highest content of polyphenols and flavonoids, along with the highest antioxidant activity, followed by the methanolic extract (E2) and, lastly, by the aqueous extract (E1) (Table 1 and Table 2, Figure 2). Our results are in line with previous studies, suggesting that the bioactive compound content varies upon altitude, habitat type, and site conditions [24,25,26]. For reference, our hydro-methanolic extract presented 21.68 mg GAE/g dw total polyphenols and 8.41 mg QE/g dw flavonoids, while a similar study on bilberries from another region of Romania reported a total polyphenolic content of 34,7 - 41,9 mg GAE/g dw [32]. Similarly, a study from Turkey revealed total polyphenols of 20.06 mg GAE/g dw and total flavonoids of 2.67 mg QE/g dw [33], while another study from Germany had total polyphenols of 29.7 mg GAE/g dw, and total flavonoids of 13.5 mg QE/g dw [34].

The chemo-mapping of our extracts offers new insights into their composition, as to our knowledge, the previously reported bilberry extracts were highly focused on the anthocyanin content [2,3,4,5,6], rather than the complete phytochemical profile (Table 1). The identified chemical classes (Figure 2) are in agreement with reported composition of *Vaccinium myrtillus* L. extracts (Table S5) [35,36,37,38,39,40,41,42,43,44]. Moreover, they are in accordance with reported composition of the bilberry fruit, based on the report of the European Medicines Agency [35].

Noteworthy, the polyphenol and flavonoid content decrease with the increase in the degree of fruit ripening [35]. Oppositely, anthocyanosides—more than half of the molecules giving the color in *Vaccinium* spp.—increase in concentration when the fruit approaches maturation [35]. Carboxylic acids, vitamins, and miscellaneous classes in our samples are highly similar in composition to those reported in previous studies [35]. Regarding alkaloids and iridoids, their presence is generally scarce, especially in ripe fruit, with as little as one and two representatives [35], which is in accordance with our findings.

Approximately 40% of the compounds identified in our extracts have not been previously reported in *Vaccinium* spp., while 3 phytochemicals (cinnamtannin B1, cinnamtannin D1, and quercetin-3-*O-*galactoside) were only described in other species, and not for *Vaccinium myrtillus* L. (Table S5; Table S6—superscripts ^A^, ^B^, ^C^) [35,36,37,38,39,40,41,42,43,44]. Regarding the substances not reported earlier, it is, however, important to mention that the free amino acids presented in our study pertain to this category. This might be due to a mainly anthocyanin focused bilberry specific research, omitting, hence, the free amino acid class of compounds, though the later might confer substantial nutritional values to the bilberry extracts.

**Figure 6:**
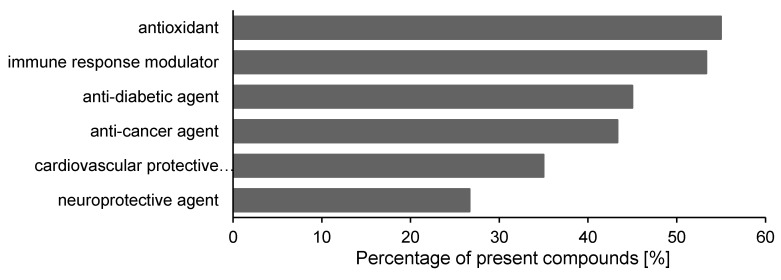
Health benefits of the compounds identified in the three extract formulations, aqueous, methanolic, and hydro-methanolic. Only health promoting effects shown by more than 20% of the phytochemicals are presented. This figure is based on the summarized information in Table S6 [45,46,47,48,49,50,51,52,53,54,55,56,57,58,59,60,61,62,63,64,65,66,67,68,69,70,71,72,73,74,75,76,77,78,79,80,81,82,83,84,85,86,87,88,89,90,91,92,93,94,95,96,97,98,99,100,101,102,103,104,105,106,107,108,109,110,111,112,113,114,115,116,117,118,119,120,121,122,123,124,125,126,127,128,129,130,131,132,133,134,135,136,137,138,139,140,141,142,143,144,145,146,147,148,149,150,151,152,153,154,155,156,157,158,159,160,161,162,163,164,165,166,167,168,169,170,171,172,173,174,175,176,177,178,179,180,181,182,183,184,185,186,187,188,189,190,191,192,193,194,195,196,197,198,199,200,201,202,203,204,205,206,207,208,209,210,211,212,213,214,215,216,217,218,219,220,221,222,223,224,225,226,227,228,229,230,231,232,233,234,235,236,237,238,239,240,241,242,243,244,245,246,247,248,249,250,251,252,253,254,255,256,257,258,259,260,261].

Furthermore, we summarized the already reported biological effects for each phytochemical identified in the studied bilberry extracts (Figure 6, Table S6) [45,46,47,48,49,50,51,52,53,54,55,56,57,58,59,60,61,62,63,64,65,66,67,68,69,70,71,72,73,74,75,76,77,78,79,80,81,82,83,84,85,86,87,88,89,90,91,92,93,94,95,96,97,98,99,100,101,102,103,104,105,106,107,108,109,110,111,112,113,114,115,116,117,118,119,120,121,122,123,124,125,126,127,128,129,130,131,132,133,134,135,136,137,138,139,140,141,142,143,144,145,146,147,148,149,150,151,152,153,154,155,156,157,158,159,160,161,162,163,164,165,166,167,168,169,170,171,172,173,174,175,176,177,178,179,180,181,182,183,184,185,186,187,188,189,190,191,192,193,194,195,196,197,198,199,200,201,202,203,204,205,206,207,208,209,210,211,212,213,214,215,216,217,218,219,220,221,222,223,224,225,226,227,228,229,230,231,232,233,234,235,236,237,238,239,240,241,242,243,244,245,246,247,248,249,250,251,252,253,254,255,256,257,258,259,260,261]. More than half of the characterized substances are reported in literature to behave like antioxidant agents. Such a category includes free radical scavengers; antilipid peroxidation agents; and molecules that inhibit or deter free radical formation, protect the target, or repair the damage produced due to oxidative stress (Table S6). The pronounced presence of various phytonutrients with antioxidant function strongly indicates that the bilberry extracts as a whole might also aid in the reduction in oxidative stress, despite the fact that the implicated coping mechanisms at cellular level are currently scarcely documented. Some already existing research data accentuate the antioxidant feature of bilberry extracts largely attributed to the rich anthocyanin content of bilberries [2,3,4,5,6,7]. Moreover, the in vitro antioxidant property of the assessed three bilberry extracts is further substantiated by the DPPH free radical scavenging assay, suggesting once again the superiority of the hydro-methanolic extract (E3) over the other formulations (Table 2).

Besides phytonutrients with antioxidant features, many others also had immune response modulator, antidiabetic, anticancer, cardiovascular protective, and neuroprotective properties (Figure 6). Interestingly, some bilberry extract-specific phytonutrients, when assessed individually using different experimental setups, have been shown to generate multiple in vivo effects. Therefore, the investigation of a bilberry extract associated with biological functions calls for a systemic approach that will let us combine genetic, nutritional, and physiological methodologies using in vivo animal models.

In line with the abovementioned considerations, we decided to take advantage of a *Drosophila melanogaster*-based nutritional genetic in vivo model for which normal media and high-sugar types of diets generated diabetic modifications could be assessed upon bilberry extract supplementation. Among the in vivo experiments, the most compellingantidiabetic rescue effect of the bilberry extract is related to the hemolymph trehalose levels that are restored to the levels of normal diet controls upon addition of current hydro-methanolic bilberry extract (E3) to the high-sugar diet (Figure 2, Table S8). When reared on high-sugar media, the level of hemolymph trehalose reached an average of 943 mg/L, while upon addition of the hydro-methanolic extract (E3), the level of hemolymph trehalose fell to an average of 447 mg/L, comparable to the levels in control normal diet averaging 457 mg/L. Similarly, Musselmann and colleagues demonstrated an approximate 2-fold increase in hemolymph trehalose levels induced by a high-sugar diet [16]; such an observation would strengthen our claim for the rescue effect exhibited by the hydro-methanolic bilberry extract (E3).

Furthermore, the hydro-methanolic bilberry extract (E3) was tested in the abovementioned dietary conditions regarding its putative rescue effects that might compensate for the high-sugar diet generated diabetic carbohydrate and lipid metabolisms, inflammation, neurodegeneration, and other Met-S associated pathologies. In this context, we chose to study the genetic control of neurohumoral regulation by assessing the mRNA expression level of several genes encoding for neuropeptides. Some of the genes in question were selected based on previously conducted studies of high-sugar diet experiments in *Drosophila* [16,17,18].

The *Drosophila* insulin-like proteins (Ilps) are released in response to high levels of circulating sugar, while a glucagon-like molecule, the adipokinetic hormone (AKH), is synthetized in response to low levels of circulating sugar [262,263,264,265]. Our result shows very slight differences, less than 15%, between the gene expression of *Ilp*(s) in flies reared for 12 days on a high-sugar diet, as compared to those fed on a normal diet control. In the case of hydro-methanolic bilberry extract (E3) supplementation, the *Ilp*(s) expression levels are observed to be drastically reduced for *Ilp2* and *Ilp5* reaching about 20% of the control levels, while they are about 50% increased for *Ilp6*. This corresponds to the observed activity of Ilps, as Ilp6 is an insulin receptor agonist which has been shown in other contexts to work in opposition to the major circulating Ilps [266,267]. Upon starvation, Ilp6 is induced in the fat body and acts on the insulin-producing cells to downregulate *Ilp2* and *Ilp5* [268]. In our study, the expression of *Akh* is raised by the chronic exposure to elevated dietary sugar, with a foldchange comparable to the previously reported findings [16,17]. As noted also by previous authors, the increase in expression levels of *Akh* could lead to a paradoxal mobilization of stored fat and carbohydrates [16,265], without affecting metabolic rate but reducing food intake [265]. The rescue effect of the bilberry extract is also remarkedly pronounced in the case of *Akh* gene, whose expression is reduced at about 95% as compared to the appropriate control, suggesting that the bilberry extract could compensate for the high-sugar diet affected *Ilps* and *Akh* genes.

InR, the fruit fly insulin receptor, mediates functions similar to those of insulin and insulin-like growth factor receptors in humans [269]. In our study, it presents a rescue effect upon bilberry extract supplementation of the high sugar diet, with above 60% overexpression observed when adult flies are reared on a high-sugar diet alone, and reduced overexpression to about 35% in the case of hydro-methanolic bilberry extract (E3) supplementation of the high-sugar diet as compared to the respective controls. The expression levels of *InR* oppose those of Ilp2 and Ilp5, corresponding to previously described trends, and reinforcing the suitability of *Drosophila* to study action mechanisms generated by bilberry in relation to diabetes, insulin resistance, and other associated disease [270].

Among the assessed genes, *AstA*, *AstC*, *CCHa2*, *Nplp2*, and *Nplp3* are mainly expressed in the *Drosophila* gut, and the expression levels of these enteroendocrine peptides might fluctuate, depending on the nutritional state and dietary conditions [271]. Our results show a rescue effect upon addition of the hydro-methanolic extract (E3) to the diet for the gene expression levels of *AstA*, *AstC*, and *CCHa2*. The most dramatic foldchanges in expression levels were observed for *Nplp2* and *Nplp3*, reaching above 20-fold increases upon extract supplementation to the high-sugar diet as compared to their controls. However, the exact functional relevance of Nplp2 and Nplp3 as neuropeptides remains elusive.

Starting 24 h from eclosion, only 2 residual neurons in *Drosophila* express *CCAP* (crustacean cardioactive peptide), a gene extensively studied in the context of ecdysis [272,273], recently proved to signal for food intake and regulate the triglyceride metabolism. The adult flies lacking the CCAP peptide had significantly lower triglyceride levels [274]. This correlates with the expression levels observed by us upon supplementation of the high-sugar diet with hydro-methanolic bilberry extract (E3). The *CCAP* expression levels shows a 2-fold decrease for the high-sugar diet, while a 30-fold decrease is seen for the extract-supplemented high-sugar diet, as compared to their appropriate controls. Our observations related to the expression of the *CCAP* gene suggest its possible implication in the triglyceride lowering effect of the bilberry extract.

Npc2g is a neuropeptide that plays a role in the cholesterol breakdown, absorption, and trafficking, along with Hr96 [275,276]. In our study, a counteracting rescue effect on the gene expression of *Npc2g* is observed, with a 3-fold increase in the case of hydro-methanolic bilberry extract (E3) supplementation to the high-sugar diet, while presenting a 3-fold decrease in the case of the high-sugar diet alone, as compared to their appropriate controls. The *Npc2g* specific gene expression profile indicates the bilberry extract possible implication in cholesterol homeostasis. However, no significant modifications could be observed in the case of the expression levels of *Hr96* gene.

The *SIFa* gene has been observed to have a function in food uptake by enhancing the taste-guided and odor-guided appetitive behavior [277]. No change is observed in our experiments when comparing the expression levels of the *SIFa* gene in flies having a high-sugar diet or a normal diet control regimen; however, there is a 4-fold decrease in its expression observed upon hydro-methanolic bilberry extract (E3) addition to the high-sugar diet. However, the effect of this modification on the *Drosophila* metabolism is still unclear.

The *pdf* is another gene involved in the feeding behavior. While it is not described as regulating the carbohydrate or fat metabolisms in *Drosophila*, its function as a major synchronization and output factor of the circadian clock [278] is well studied as affecting the timing of sleep and feeding [279,280], with AstA-expressing PLP neurons as a downstream target of the clock output factor PDF [281]. While a rescue effect of of the hydro-methanolic bilberry extract (E3) is observed for the *AstA* gene expression levels, the same trend of downregulation is observed for the expression of *pdf*, as its expression changes only in the case of extract-supplemented high-sugar diet, decreasing 3-fold as compared to the respective control.

Despite the undetermined function of CNMa protein [282], it is noteworthy that the *CNMa* gene expression patterns under the test conditions are similar to those of *pdf* and *SIFa*, with very slight variations caused by the high-sugar diet, and about 3-fold decrease measured upon addition of hydro-methanolic bilberry extract (E3).

The tested transcript levels of *Thor* gene were previously considered as a readout of the peripheral insulin signaling [283]. *Thor* is a target of the evolutionarily conserved transcription box Forkhead, which is repressed by insulin signaling [284,285]. Hence, *Thor* is repressed by the active peripheral insulin signaling [284,285], and increased brain insulin levels are expected to reduce *Thor* expression levels. In contrast to this expectation, it was observed that *Thor* transcript levels are unaffected in Akh loss-of-function mutants, suggesting that the peripheral insulin signaling is impaired in response to AKH deficiency [283]. On the other hand, our results indicate no significant changes in the *Thor* expression levels as compared to the extent of *Akh* expression pattern. The high-sugar diet with or without extract supplementation experiments did not provide us relevant functional cues in relation to the *Akh*-*Thor* previously suggested interdependence on a normal diet.

Different from the *Thor* expression pattern, the transcription levels of *tobi,* another insulin target that encodes for a putative amyloglucosidase [283], shows a rescue effect due to the hydro-methanolic bilberry extract (E3) supplementation of the high-sugar diet as compared to the controls. Our observations are in contrast with the findings of Galikova and colleagues, where *Akh* over-expression led to the non-significant modification of the *tobi* mRNA levels [283]. Taken together, our data suggest that the expression pattern of the *Akh*-*Thor* and *tobi* genes seems to depend considerably on dietary conditions.

The gene called *inwardly rectifying potassium channel1 (Irk1)* is responsible for transepithelial ion transport in the *Drosophila* renal tubule and is additive to Na^+^/K^+^-ATPase-dependent pathways [286]. Its main functions are iono- and osmoregulation, and along with the intricate links with sugar and fat metabolism, in our study, there is a rescue effect exhibited by extract supplementation under these test conditions, where the *Irk* expression is downregulated by high-sugar diet alone, whilst it is overexpressed upon hydro-methanolic bilberry extract (E3) supplementation.

Insulin-degrading enzyme (IDE) is a neutral zinc and thiol-dependent metallopeptidase, for which increasing evidence suggests its dysfunction leads to pathogenesis of type II diabetes [287,288,289,290]. Moreover, the enzymatic activity of Ide targets, besides insulin, also other hormones, suggesting a complex contribution of Ide to the regulation of carbohydrate metabolism [291]. In our analysis, the high-sugar diet alone induces the downregulation of *Ide* expression, while supplementation of bilberry extract enhances the downregulation under tested conditions.

The gene *Eip75B* plays a role in steroid signaling, regulating the circadian rhythm and protecting the central clock against environmental stressors in *Drosophila* [292]. It was previously proven that nutrition rich in sugars, used as a stressor, can induce arrhythmic or weak circadian behavior upon *Eip75B* knockdown [292]. Hence, it could be hypothesized that the 2.75-fold upregulation of the *Eip75B* expression on our high-sugar diet supplemented with hydro-methanolic bilberry extract (E3) is either a result of additional stress induced by the extract, or a boost for the expression of *Eip75B* meant to help in compensating for the nutritional stressor.

The gene *CG15618* has been identified as the *Drosophila* ortholog of THADA (Thyroid adenoma-associated protein homolog) [293], the latter being identified as one of the top risk loci for type 2 diabetes in human genome-wide association studies [294]. In *Drosophila, the CG15618* acts like a metabolic regulator that would match fat storage and heat production. In our experiments, the obese and hyperphagic phenotypes specific for the reduced expression of *CG15618* were not recapitulated (data not shown). Moreover, the similar level of downregulation of *CG15618* expression, seen for both the bilberry extract-supplemented high-sugar diet and the high-sugar diet alone, would indicate that the applied dietary conditions do not affect or interfere with the functions of the gene in question.

The Usp (ultraspiracle) protein belongs to a family of nuclear hormone receptors that are ligand-inducible transcription regulators [295]. The *usp* gene is the insect ortholog of the vertebrate retinoid X receptor (RXR) [296], similarly to which it heterodimerizes with nuclear receptors to form active receptor complexes. Its main interaction partner is the ecdysone receptor (EcR), the receptor of ecdysteroids, insect steroid hormones that control development, reproduction, together with starvation response [297]. It is unclear what function it holds in the context of our experimental setup; however, it is observed that *usp* expression is about 30% upregulated for flies reared on a high-sugar diet, both with and without bilberry extract supplementation.

Taken together, the application of the high-sugar diet and *Drosophila-*based experimental approach to study the bilberry extract induced in vivo effects looks rather promising and recapitulates not only metabolic syndrome-related aspects but could also give a sneak peek at other diet influenceable pathologies. Besides the bilberry relatedantidiabetic aspects of neurohormonal regulation, it would be equally important to look at the genetic control of antioxidant andanticancer effects upon dietary conditions that could widen or restrict the preventive/therapeutic applicability of bilberries. For example, from the standpoint of colorectal cancer, the 3rd most common type of cancer in the world and the 2nd most responsible for the number of cancer-related deaths worldwide [298], the elevated levels of *Thor* expression represent a poor prognosis indicator, and hence it is considered a potential colorectal cancer biomarker [299]. We were able to demonstrate for *Drosophila* that the high-sugar diet alone or supplemented with bilberry extract could not significantly modify the expression levels of *Thor*, suggesting that the relevance of *Thor* expression level and the peripheral insulin sensitivity connection has to be carefully reconsidered in the context of future colorectal cancer studies. Noteworthy, the above presented picture would become even more complicated if we were to consider the *Akh*-*Thor* connections too.

## 5. Conclusions

In conclusion, our study on bilberry revealed a complex phytonutrient profile that could be associated withantidiabetic effects, such as efficiently reducing the haemolymph trehalose levels using a *Drosophila melanogaster* based high-sugar diet model. Moreover, the bilberry induced beneficial effect is substantiated by reversing the affected expression level of some genes implicated in the neuro-hormonal control of the high-sugar diet induced pathologies. Our results also suggest that the beneficial effects of diet-based supplementation of bilberry would generate multiple compensatory mechanisms as seen for *Drosophila* genes such as *InR, Akh, AstA, AstC, Irk, Npc2g,* and *CCHa2*. Defining the interdependence of the abovementioned genes and assessing the whole genome response upon diet supplementation with bilberry requires further transcriptomic studies that undoubtedly could generate rather advanced interactomes explaining the complexity of functional consequences. In a larger context, our data contribute to a better understanding of the metabolic syndrome associated pathologies, such as type II diabetes and various cancers [20,21,22,23,298], alongside the associated genetic and environmental risk factors, and the elucidation of the bilberry-based preventive/therapeutic diet-induced genetic and physiological consequences.

## Figures and Tables

**Table 1:** Summary of the putative identification of phytonutrients in the extracts, with compounds pertaining to each extract (E1, E2, and E3) marked ✓—if present and ✗—if absent. Compounds are organized by chemical classification.

Chemical Classification	Putative Identification	E1	E2	E3
Flavonoids	Ampelopsin (Ampeloptin, Dihydromyricetin)	✗	✗	✓
	Avicularin (Quercetin-3-*O-*arabinofuranoside, Fenicularin)	✓	✓	✓
	Catechin or Epicatechin-*O-*hexoside	✓	✓	✓
	Cinnamtannin B1	✓	✓	✓
	Cinnamtannin D1	✓	✓	✓
	Cyanidin-3-*O-*arabinoside	✓	✓	✓
	Cyanidin-3-*O-*glucoside (Kuromanin, Asterin, Chrysanthemin)	✓	✓	✓
	Cyanidin-3-*O-*sambubioside (Sambicyanin, Gossypicyanin)	✓	✓	✓
	Cyanidin-*O-*(coumaroyl)hexoside	✗	✓	✓
	Delphinidin-3-*O-*arabinoside	✓	✓	✓
	Delphinidin-3-*O-*galactoside (Empetrin)	✓	✓	✓
	Delphinidin-*O-*(pentosyl)hexoside	✗	✓	✓
	Epicatechin	✓	✓	✓
	Epigallocatechin	✓	✓	✓
	Gallocatechin	✓	✓	✓
	Hyperoside (Quercetin-3-*O-*galactoside, Hyperin)	✓	✓	✓
	Idaein (Idein, Cyanidin-3-*O-*galactoside)	✓	✓	✓
	Isoquercitrin (Hirsutrin, Quercetin-3-*O-*glucoside)	✓	✓	✓
	Isorhamnetin-*O-*glucuronide	✓	✓	✓
	Kaempferol-3-*O-*glucuronide	✓	✓	✓
	Laricitrin (Myricetin-3′-*O-*methyl ether)	✓	✓	✓
	Laricitrin-*O-*hexoside	✓	✓	✓
	Malvidin-3-*O-*arabinoside	✓	✓	✓
	Malvidin-*O-*(coumaroyl)hexoside	✗	✓	✓
	Malvidin-*O-*hexoside	✓	✓	✓
	Myricetin	✗	✓	✓
	Myricetin-3-*O-*arabinoside	✓	✓	✓
	Myricetin-*O-*hexoside	✓	✓	✓
	Myricetin-*O-*pentoside isomer	✓	✓	✓
	Naringenin	✓	✓	✓
	Naringenin chalcone	✗	✓	✓
	Pentahydroxyflavone (Hypolaetin, Quercetin, Tricetin)	✓	✓	✓
	Peonidin-3-*O-*arabinoside	✓	✓	✓
	Peonidin-*O-*(coumaroyl)hexoside	✗	✓	✓
	Peonidin-*O-*(pentosyl)hexoside	✗	✓	✗
	Peonidin-*O-*hexoside	✓	✓	✓
	Peonidin-*O-*pentoside isomer	✗	✓	✗
	Petunidin-3-*O-*arabinoside	✓	✓	✓
	Petunidin-3-*O-*galactoside	✓	✓	✓
	Petunidin-*O-*(pentosyl)hexoside	✗	✓	✗
	Procyanidin B1 or B3	✓	✓	✓
	Prunin (Naringenin 7-*O-*glucoside)	✓	✓	✓
	Quercetin-3-*O-*[3-Hydroxy-3-methylglutaroyl-(→4)-rhamnoside]	✓	✓	✓
	Quercetin-3-*O-*glucuronide	✓	✓	✓
	Quercetin-*O-*(coumaroyl)hexoside	✗	✓	✓
	Quercetin-*O-*rhamnoside-*O-*pentoside	✓	✓	✓
	Quercitrin (Quercetin-3-*O-*rhamnoside)	✓	✓	✓
	Syringetin-*O-*hexoside	✓	✓	✓
	Trihydroxyflavanone	✗	✓	✓
Amino acids	2-Aminoadipic acid	✗	✓	✓
	Arginine	✓	✓	✓
	Asparagine	✗	✓	✓
	Glutamic acid	✓	✗	✓
	Histidine	✓	✗	✓
	Isoleucine or Leucine	✗	✓	✓
	Lysine	✓	✗	✓
	Phenylalanine	✓	✓	✓
	Threonine	✓	✓	✓
	Tryptophan	✓	✓	✓
	Tyrosine	✓	✓	✓
	γ-Aminobutyric acid (GABA)	✓	✗	✗
Polyphenols	Caffeoylshikimic acid	✓	✓	✓
	Chlorogenic acid (3-*O-*Caffeoylquinic acid)	✓	✓	✓
	Coumaroylquinic acid	✓	✓	✓
	Coumaroyl-shikimate	✓	✓	✓
	Feruloylquinic acid	✓	✓	✓
	Gallic acid (3,4,5-Trihydroxybenzoic acid)	✓	✓	✓
Carboxylic acids	4-Coumaric acid	✓	✓	✓
	Caffeic acid	✓	✓	✓
	Dihydroxy-methoxybenzoic acid	✗	✓	✓
	Dimethoxy-hidroxycinnamic acid (Sinapic acid)	✓	✓	✓
	Ferulic acid	✓	✓	✓
Vitamins	Adenine	✗	✓	✓
	Nicotinamide	✓	✓	✓
	Nicotinic acid (B3)	✗	✓	✓
	Pantothenic acid (B5)	✗	✗	✓
Terpenoids	Abscisic acid (ABA)	✓	✓	✓
Sugars	Saccharic acid	✓	✓	✓
Lactones	Gulonic acid γ-lactone or δ-Gluconic acid δ-lactone	✓	✓	✓
Esters	Methyl gallate	✓	✓	✓
Alkaloids	Choline	✓	✗	✗
Iridoids	7-Deoxyloganic acid	✗	✓	✓
Miscellaneous	4-Methoxycinnamaldehyde	✗	✗	✓
	5-Hydroxymethyl-2-furaldehyde	✓	✓	✓
	5-Methyl-2-furaldehyde	✓	✓	✓
	N-(2-Phenylethyl)acetamide	✓	✗	✗
	Phytosphingosine	✗	✓	✗
	Vanillin	✗	✓	✓

**Table 2:** Total polyphenolic and flavonoid content, along with antioxidant activity of the three formulations of the bilberry ripe fruit extracts.

Extract Formulation	Total Polyphenols [mg GAE/g dw]	Total Flavonoids [mg QE/g dw]	Antioxidant Activity [%]
Aqueous extract (E1)	12.48 ± 0.13	3.88 ± 0.11	50.87 ± 0.12
Methanolic extract (E2)	20.42 ± 0.17	7.27 ± 0.12	77.32 ± 0.09
Hydro-methanolic extract (E3)	21.68 ± 0.19	8.41 ± 0.11	78.03 ± 0.16

## Supplementary Materials

The following are available online at https://www.mdpi.com/2076-3921/9/11/1067/s1.
