# Supplementary material for: Bilberry (Vaccinium myrtillus L.) Extracts Comparative Analysis Regarding Their Phytonutrient Profiles, Antioxidant Capacity along with the In Vivo Rescue Effects Tested on a Drosophila melanogaster High-Sugar Diet Model
Source: Antioxidants (Basel). 2020 Oct 30;9(11):1067. doi: 10.3390/antiox9111067 (PMC7694118; doi:10.3390/antiox9111067)
Supplement: Supplementary file 1 [file antioxidants-09-01067-s001.pdf]

antioxidants

MDPI

Supplementary Materials

Table S1. Chemical composition of the ripe bilberry fruit aqueous extract (E1) determined using UHPLC-ESI-MS/MS. The chemical classification of the compounds was assigned for all compounds, along with the presented molecular formula, retention time (RT), radical molecular cations (M [+]) and anions (M[-]).Fragmentation pattern was omitted and can be found in **Table S4**.

No.	Chemical classification	Compound	Molecular formula	RT	M [+]	M [-]
1	Amino acid	Lysine	C6H14N2O2	1.08	147.11336	
2	Alkaloid	Choline	C5H14NO	1.16	104.10754	
3	Amino acid	Arginine	C6H14N4O2	1.16	175.11951	
4	Amino acid	Histidine	C6H9N3O2	1.16	156.07731	
5	Amino acid	γ-Aminobutyric acid	C4H9NO2	1.19	104.07116	
6	Amino acid	Glutamic acid	C5H9NO4	1.20	148.06099	
7	Amino acid	Threonine	C4H9NO3	1.20	120.06607	
8	Sugar	Saccharic acid	C6H10O8	1.20		209.02975
9	Miscellaneous	5-Hydroxymethyl-2-furaldehyde	C6H6O3	1.24	127.03952	
10	Lactone	Gulonic acid γ-lactone or δ-Gluconic acid δ-lactone	C6H10O6	1.27	179.05557	
11	Miscellaneous	5-Methyl-2-furaldehyde	C6H6O2	1.29	111.04461	
12	Vitamin	Nicotinic acid (Niacin,B3)	C6H5NO2	1.44	124.03986	
13	Amino acid	Tyrosine	C9H11NO3	1.71	182.08172	
14	Carboxylic acid	Gallic acid (3,4,5-Trihydroxybenzoic acid)	C7H6O5	2.26		169.01370
15	Amino acid	Phenylalanine	C9H11NO2	3.19	166.08681	
16	Flavonoid	Gallocatechin	C15H14O7	4.23		305.06613
17	Amino acid	Tryptophan	C11H12N2O2	8.12	205.09771	
18	Ester	Methyl gallate	C8H8O5	8.42		183.02935
19	Flavonoid	Procyanidin B1 or B3	C30H26O12	11.85	579.15025	
20	Flavonoid	Catechin	C15H14O6	13.20		289.07121
21	Flavonoid	Epigallocatechin	C15H14O7	13.47		305.06613
22	Flavonoid	Catechin or Epicatechin-O-hexoside	C21H24O11	14.00		451.12404
23	Polyphenol	Chlorogenic acid (3-O-Caffeoylquinicacid)	C16H18O9	14.12	355.10291	
24	Carboxylic acid	Caffeic acid	C9H8O4	14.28		179.03444
25	Flavonoid	Procyanidin B1 or B3	C30H26O12	15.15	579.15025	
26	Flavonoid	Cinnamtannin D1	C45H36O18	15.24		863.18234
27	Carboxylic acid	Dimethoxy-hidroxycinnamic acid	C11H12O5	15.60	225.07630	
28	Flavonoid	Cinnamtannin B1	C45H36O18	15.78		863.18234
29	Flavonoid	Delphinidin-3-O-galactoside (Empetrin)	C21H21O12	16.17	465.10330	
30	Polyphenol	Coumaroylquinic acid isomer 1	C16H18O8	16.83		337.09235
31	Flavonoid	Epicatechin	C15H14O6	17.02		289.07121
32	Flavonoid	Delphinidin-3-O-arabinoside	C20H19O11	17.03	435.09274	
33	Flavonoid	Idaein (Idein, Cyanidin-3-O-galactoside)	C21H21O11	17.06	449.10839	
34	Flavonoid	Cyanidin-3-O-glucoside (Kuromanin, Asterin, Chrysanthemin)	C21H21O11	17.40	449.10839	

No.	Chemical classification	Compound	Molecular formula	RT	M [+]	M [-]
35	Flavonoid	Cyanidin-3-O-sambubioside (Sambicyanin, Gossypicyanin)	C26H29O15	17.71	581.15065	
36	Carboxylic acid	4-Coumaric acid	C9H8O3	17.75		163.03952
37	Flavonoid	Cyanidin-3-O-arabinoside	C20H19O10	17.84	419.09783	
38	Polyphenol	Caffeoylshikimic acid	C16H16O8	17.93		335.07670
39	Flavonoid	Petunidin-3-O-galactoside	C22H23O12	17.97	479.11895	
40	Polyphenol	Feruloylquinic acid	C17H20O9	17.97		367.10291
41	Flavonoid	Petunidin-3-O-arabinoside	C21H21O11	18.36	449.10839	
42	Flavonoid	Pelargonidin-O-hexoside	C21H21O10	18.47	433.11347	
43	Flavonoid	Peonidin-O-hexoside	C22H23O11	18.89	463.12404	
44	Flavonoid	Malvidin-O-hexoside	C23H25O12	19.04	493.13460	
45	Polyphenol	Coumaroylquinic acid isomer 2	C16H18O8	19.16		337.09235
46	Carboxylic acid	Ferulic acid	C10H10O4	19.29		193.05009
47	Flavonoid	Peonidin-3-O-arabinoside	C21H21O10	19.41	433.11347	
48	Flavonoid	Malvidin-3-O-arabinoside	C22H23O11	19.80	463.12404	
49	Miscellaneous	N-(2-Phenylethyl)acetamide	C10H13NO	20.10	164.10754	
50	Polyphenol	Coumaroyl-shikimate	C16H16O7	20.34		319.08178
51	Flavonoid	Myricetin-O-hexoside	C21H20O13	20.86		479.08257
52	Flavonoid	Myricetin-3-O-arabinoside	C20H18O12	20.97		449.07201
53	Flavonoid	Myricetin-O-pentoside isomer	C20H18O12	21.81		449.07201
54	Flavonoid	Prunin (Naringenin 7-O-glucoside)	C21H22O10	22.36	435.12912	
55	Flavonoid	Hyperoside (Quercetin-3-O-galactoside, Hyperin)	C21H20O12	22.64		463.08765
56	Flavonoid	Quercetin-3-O-glucuronide	C21H18O13	22.66		477.06692
57	Flavonoid	Isoquercitrin (Hirsutrin, Quercetin-3-O-glucoside)	C21H20O12	22.87		463.08765
58	Flavonoid	Laricitrin (Myricetin-3'-O-methyl ether)	C16H12O8	23.19	333.06105	
59	Flavonoid	Laricitrin-O-hexoside	C22H22O13	23.20		493.09822
60	Flavonoid	Avicularin (Quercetin-3-O-arabinofuranoside, Fenicularin)	C20H18O11	23.47		433.07709
61	Flavonoid	Quercitrin (Quercetin-3-O-rhamnoside)	C21H20O11	24.43	449.10839	
62	Flavonoid	Kaempferol-3-O-glucuronide	C21H18O12	24.58		461.07200
63	Flavonoid	Quercetin-O-rhamnoside-O-pentoside	C26H28O15	24.61	581.15065	
64	Flavonoid	Syringetin-O-hexoside	C23H24O13	24.96		507.11387
65	Flavonoid	Isorhamnetin-O-glucuronide	C22H20O13	25.12		491.08257
66	Terpenoid	Abscisic acid	C15H20O4	25.40		263.12834
67	Flavonoid	Quercetin-3-O-[3-Hydroxy-3-methylglutaroyl-(→4)-rhamnoside]	C27H28O15	26.82		591.13500
68	Flavonoid	Pentahydroxyflavone	C15H10O7	27.01		301.03483
69	Flavonoid	Naringenin	C15H12O5	27.25		271.06065

Table S2. Chemical composition of the ripe bilberry fruit methanolic extract (E2) determined using UHPLC-ESI-MS/MS. The chemical classification of the compounds was assigned for all compounds, along with the presented molecular formula, retention time (RT), radical molecular cations (M [+]) and anions (M[-]).Fragmentation pattern was omitted and can be found in **Table S4**.

No.	Chemical classification	Compound	Molecular formula	RT	M [+]	M [-]
1	Amino acid	Arginine	C6H14N4O2	1.15	175.11951	
2	Amino acid	Threonine	C4H9NO3	1.20	120.06607	
3	Amino acid	Asparagine	C4H8N2O3	1.21	133.06132	
4	Amino acid	2-Aminoadipic acid	C6H11NO4	1.22	162.07664	
5	Sugar	Saccharic acid	C6H10O8	1.22		209.02975
6	Vitamin	Adenine	C5H5N5	1.22	136.06233	
7	Vitamin	Nicotinic acid (Niacin,B3)	C6H5NO2	1.22	124.03986	
8	Miscellaneous	5-Hydroxymethyl-2-furaldehyde	C6H6O3	1.24	127.03952	
9	Miscellaneous	5-Methyl-2-furaldehyde	C6H6O2	1.25	111.04461	
10	Lactone	Gulonic acid γ-lactone or δ-Gluconic acid δ-lactone	C6H10O6	1.28	179.05557	
11	Vitamin	Nicotinamide	C6H6N2O	1.44	123.05584	
12	Amino acid	Tyrosine	C9H11NO3	1.71	182.08172	
13	Amino acid	Isoleucin or Leucin	C6H13NO2	1.83	132.10246	
14	Carboxylic acid	Gallic acid (3,4,5-Trihydroxybenzoic acid)	C7H6O5	2.26		169.01370
15	Amino acid	Phenylalanine	C9H11NO2	3.02	166.08681	
16	Flavonoid	Gallocatechin	C15H14O7	4.23		305.06613
17	Amino acid	Tryptophan	C11H12N2O2	7.98	205.09771	
18	Ester	Methyl gallate	C8H8O5	8.46		183.02935
19	Carboxylic acid	Dihydroxy-methoxybenzoic acid	C8H8O5	11.19		183.02935
20	Flavonoid	Catechin	C15H14O6	13.19		289.07121
21	Flavonoid	Epigallocatechin	C15H14O7	13.45		305.06613
22	Flavonoid	Catechin or Epicatechin-O-hexoside	C21H24O11	13.94		451.12404
23	Polyphenol	Chlorogenic acid (3-O-Caffeoylquinic acid)	C16H18O9	14.09	355.10291	
24	Carboxylic acid	Caffeic acid	C9H8O4	14.25		179.03444
25	Flavonoid	Procyanidin B1 or B3	C30H26O12	15.11	579.15025	
26	Flavonoid	Cinnamtannin D1	C45H36O18	15.20		863.18234
27	Miscellaneous	Vanillin	C8H8O3	15.50	153.05517	
28	Carboxylic acid	Dimethoxy-hidroxycinnamic acid	C11H12O5	15.56	225.07630	
29	Flavonoid	Cinnamtannin B1	C45H36O18	15.75		863.18234
30	Flavonoid	Delphinidin-3-O-galactoside (Empetrin)	C21H21O12	15.90	465.10330	
31	Flavonoid	Delphinidin-O-(pentosyl)hexoside	C26H29O16	16.15	597.14556	
32	Flavonoid	Delphinidin-3-O-arabinoside	C20H19O11	16.75	435.09274	
33	Flavonoid	Idaein (Idein, Cyanidin-3-O-galactoside)	C21H21O11	16.76	449.10839	
34	Polyphenol	Coumaroylquinic acid isomer 1	C16H18O8	16.80		337.09235
35	Flavonoid	Epicatechin	C15H14O6	17.00		289.07121
36	Flavonoid	Cyanidin-3-O-glucoside (Kuromanin, Asterin, Chrysanthemin)	C21H21O11	17.25	449.10839	

No.	Chemical classification	Compound	Molecular formula	RT	M [+]	M [-]
37	Flavonoid	Cyanidin-3-O-sambubioside (Sambicyanin, Gossypicyanin)	C26H29O15	17.63	581.15065	
38	Flavonoid	Petunidin-O-(pentosyl)hexoside	C27H30O16	17.66	611.16066	
39	Flavonoid	Cyanidin-3-O-arabinoside	C20H19O10	17.72	419.09783	
40	Flavonoid	Petunidin-3-O-galactoside	C22H23O12	17.73	479.11895	
41	Carboxylic acid	4-Coumaric acid	C9H8O3	17.73		163.03952
42	Polyphenol	Caffeoylshikimic acid	C16H16O8	17.93		335.07670
43	Polyphenol	Feruloylquinic acid	C17H20O9	17.94		367.10291
44	Flavonoid	Petunidin-3-O-arabinoside	C21H21O11	18.35	449.10839	
45	Flavonoid	Pelargonidin-O-hexoside	C21H21O10	18.47	433.11347	
46	Flavonoid	Peonidin-O-hexoside	C22H23O11	18.86	463.12404	
47	Flavonoid	Peonidin-O-(pentosyl)hexoside	C27H30O15	18.93	595.16575	
48	Flavonoid	Malvidin-O-hexoside	C23H25O12	19.00	493.13460	
49	Polyphenol	Coumaroylquinic acid isomer 2	C16H18O8	19.15		337.09235
50	Flavonoid	Peonidin-3-O-arabinoside	C21H21O10	19.27	433.11347	
51	Carboxylic acid	Ferulic acid	C10H10O4	19.27		193.05009
52	Flavonoid	Malvidin-3-O-arabinoside	C22H23O11	19.70	463.12404	
53	Polyphenol	Coumaroyl-shikimate	C16H16O7	20.34		319.08178
54	Flavonoid	Myricetin-O-hexoside	C21H20O13	20.84		479.08257
55	Flavonoid	Myricetin-3-O-arabinoside	C20H18O12	20.96		449.07201
56	Iridoid	7-Deoxyloganic acid	C16H24O9	21.23		359.13421
57	Flavonoid	Peonidin-O-pentoside isomer	C21H21O10	21.45	433.11347	
58	Flavonoid	Myricetin-O-pentoside isomer	C20H18O12	21.80		449.07201
59	Miscellaneous	Naringenin chalcone isomer 1	C15H12O5	22.35	273.07630	
60	Flavonoid	Prunin (Naringenin 7-O-glucoside)	C21H22O10	22.35	435.12912	
61	Flavonoid	Hyperoside (Quercetin-3-O-galactoside, Hyperin)	C21H20O12	22.64		463.08765
62	Miscellaneous	Naringenin chalcone isomer 2	C15H12O5	22.65	273.07630	
63	Flavonoid	Quercetin-3-O-glucuronide	C21H18O13	22.66		477.06692
64	Flavonoid	Isoquercitrin (Hirsutrin, Quercetin-3-O-glucoside)	C21H20O12	22.89		463.08765
65	Flavonoid	Laricitrin (Myricetin-3'-O-methyl ether)	C16H12O8	23.20	333.06105	
66	Flavonoid	Laricitrin-O-hexoside	C22H22O13	23.21		493.09822
67	Flavonoid	Avicularin (Quercetin-3-O-arabinofuranoside, Fenicularin)	C20H18O11	23.48		433.07709
68	Flavonoid	Myricetin	C15H10O8	24.17		317.02974
69	Flavonoid	Cyanidin-O-(coumaroyl)hexoside isomer 1	C30H26O13	24.32	595.14517	
70	Flavonoid	Quercitrin (Quercetin-3-O-rhamnoside)	C21H20O11	24.43	449.10839	
71	Flavonoid	Kaempferol-3-O-glucuronide	C21H18O12	24.59		461.07200
72	Flavonoid	Quercetin-O-rhamnoside-O-pentoside	C26H28O15	24.63	581.15065	
73	Flavonoid	Syringetin-O-hexoside	C23H24O13	24.97		507.11387
74	Flavonoid	Isorhamnetin-O-glucuronide	C22H20O13	25.14		491.08257
75	Flavonoid	Malvidin-O-(coumaroyl)hexoside isomer 1	C32H31O14	25.22	639.17139	
76	Terpenoid	Abscisic acid	C15H20O4	25.42		263.12834
77	Flavonoid	Cyanidin-O-(coumaroyl)hexoside isomer 2	C30H26O13	25.49	595.14517	

No.	Chemical classification	Compound	Molecular formula	RT	M [+]	M [-]
78	Flavonoid	Malvidin-O-(coumaroyl)hexoside isomer 2	C32H31O14	26.23	639.17139	
79	Flavonoid	Peonidin-O-(coumaroyl)hexoside	C31H29O13	26.40	609.16082	
80	Flavonoid	Quercetin-O-(coumaroyl)hexoside	C30H26O14	26.82		609.12498
81	Flavonoid	Quercetin-3-O-[3-Hydroxy-3-methylglutaroyl-(→4)-rhamnoside]	C27H28O15	26.84		591.13500
82	Flavonoid	Pentahydroxyflavone	C15H10O7	27.00		301.03483
83	Flavonoid	Naringenin	C15H12O5	27.25		271.06065
84	Flavonoid	Trihydroxyflavanone	C15H12O5	28.28		271.06065
85	Miscellaneous	Phytosphingosine	C18H39NO3	39.88	318.30082	

Table S3. Chemical composition of the ripe bilberry fruit hydro-methanolic (1:1 water:methanol ratio) extract (E3) determined using UHPLC-ESI-MS/MS. The chemical classification of the compounds was assigned for all compounds, along with the presented molecular formula, retention time (RT), radical molecular cations (M [+]) and anions (M[-]).Fragmentation pattern was omitted and can be found in **Table S4**.

No.	Chemical classification	Compound	Molecular formula	RT	M [+]	M [-]
1	Amino acid	Lysine	C6H14N2O2	1.09	147.11336	
2	Amino acid	Arginine	C6H14N4O2	1.17	175.11951	
3	Amino acid	Histidine	C6H9N3O2	1.18	156.07731	
4	Amino acid	Asparagine	C4H8N2O3	1.21	133.06132	
5	Amino acid	Glutamic acid	C5H9NO4	1.21	148.06099	
6	Amino acid	Threonine	C4H9NO3	1.21	120.06607	
7	Vitamin	Nicotinic acid (Niacin,B3)	C6H5NO2	1.21	124.03986	
8	Sugar	Saccharic acid	C6H10O8	1.22		209.02975
9	Miscellaneous	5-Hydroxymethyl-2-furaldehyde	C6H6O3	1.24	127.03952	
10	Amino acid	2-Aminoadipic acid	C6H11NO4	1.30	162.07664	
11	Miscellaneous	5-Methyl-2-furaldehyde	C6H6O2	1.30	111.04461	
12	Lactone	Gulonic acid γ-lactone or δ-Gluconic acid δ-lactone	C6H10O6	1.30	179.05557	
13	Vitamin	Adenine	C5H5N5	1.30	136.06233	
14	Vitamin	Nicotinamide	C6H6N2O	1.43	123.05584	
15	Amino acid	Tyrosine	C9H11NO3	1.65	182.08172	
16	Amino acid	Isoleucin or Leucin	C6H13NO2	1.91	132.10246	
17	Carboxylic acid	Gallic acid (3,4,5-Trihydroxybenzoic acid)	C7H6O5	2.23		169.01370
18	Amino acid	Phenylalanine	C9H11NO2	3.15	166.08681	
19	Flavonoid	Gallocatechin	C15H14O7	4.22		305.06613
20	Vitamin	Pantothenic acid (B5)	C9H17NO5	4.99	220.11850	
21	Amino acid	Tryptophan	C11H12N2O2	8.03	205.09771	
22	Ester	Methyl gallate	C8H8O5	8.42		183.02935
23	Carboxylic acid	Dihydroxy-methoxybenzoic acid	C8H8O5	11.24		183.02935
24	Flavonoid	Procyanidin B1 or B3	C30H26O12	11.83	579.15025	
25	Flavonoid	Catechin	C15H14O6	13.21		289.07121
26	Flavonoid	Epigallocatechin	C15H14O7	13.47		305.06613
27	Flavonoid	Catechin or Epicatechin-O-hexoside	C21H24O11	14.01		451.12404
28	Polyphenol	Chlorogenic acid (3-O-Caffeoylquinic acid)	C16H18O9	14.12	355.10291	
29	Carboxylic acid	Caffeic acid	C9H8O4	14.29		179.03444
30	Flavonoid	Ampelopsin (Ampeloptin, Dihydromyricetin)	C15H12O8	14.62		319.04540
31	Flavonoid	Procyanidin B1 or B3	C30H26O12	15.13	579.15025	
32	Flavonoid	Cinnamtannin D1	C45H36O18	15.24		863.18234
33	Miscellaneous	Vanillin	C8H8O3	15.50	153.05517	
34	Carboxylic acid	Dimethoxy-hidroxycinnamic acid	C11H12O5	15.59	225.07630	
35	Flavonoid	Cinnamtannin B1	C45H36O18	15.77		863.18234
36	Flavonoid	Delphinidin-3-O-galactoside (Empetrin)	C21H21O12	15.98	465.10330	

No.	Chemical classification	Compound	Molecular formula	RT	M [+]	M [-]
37	Flavonoid	Delphinidin-O-(pentosyl)hexoside	C26H29O16	16.26	597.14556	
38	Flavonoid	Delphinidin-3-O-arabinoside	C20H19O11	16.77	435.09274	
39	Flavonoid	Idaein (Idein, Cyanidin-3-O-galactoside)	C21H21O11	16.79	449.10839	
40	Polyphenol	Coumaroylquinic acid isomer 1	C16H18O8	16.82		337.09235
41	Flavonoid	Epicatechin	C15H14O6	17.03		289.07121
42	Flavonoid	Cyanidin-3-O-glucoside (Kuromanin, Asterin, Chrysanthemin)	C21H21O11	17.38	449.10839	
43	Flavonoid	Cyanidin-3-O-sambubioside (Sambicyanin, Gossypicyanin)	C26H29O15	17.66	581.15065	
44	Carboxylic acid	4-Coumaric acid	C9H8O3	17.75		163.03952
45	Flavonoid	Cyanidin-3-O-arabinoside	C20H19O10	17.79	419.09783	
46	Flavonoid	Petunidin-3-O-galactoside	C22H23O12	17.80	479.11895	
47	Polyphenol	Caffeoylshikimic acid	C16H16O8	17.94		335.07670
48	Polyphenol	Feruloylquinic acid	C17H20O9	17.96		367.10291
49	Flavonoid	Petunidin-3-O-arabinoside	C21H21O11	18.37	449.10839	
50	Flavonoid	Pelargonidin-O-hexoside	C21H21O10	18.55	433.11347	
51	Flavonoid	Peonidin-O-hexoside	C22H23O11	18.87	463.12404	
52	Flavonoid	Malvidin-O-hexoside	C23H25O12	19.00	493.13460	
53	Polyphenol	Coumaroylquinic acid isomer 2	C16H18O8	19.17		337.09235
54	Flavonoid	Peonidin-3-O-arabinoside	C21H21O10	19.25	433.11347	
55	Carboxylic acid	Ferulic acid	C10H10O4	19.29		193.05009
56	Flavonoid	Malvidin-3-O-arabinoside	C22H23O11	19.63	463.12404	
57	Polyphenol	Coumaroyl-shikimate	C16H16O7	20.35		319.08178
58	Flavonoid	Myricetin-O-hexoside	C21H20O13	20.84		479.08257
59	Flavonoid	Myricetin-3-O-arabinoside	C20H18O12	20.97		449.07201
60	Iridoid	7-Deoxyloganic acid	C16H24O9	21.24		359.13421
61	Flavonoid	Myricetin-O-pentoside isomer	C20H18O12	21.82		449.07201
62	Miscellaneous	Naringenin chalcone isomer 1	C15H12O5	22.36	273.07630	
63	Flavonoid	Prunin (Naringenin 7-O-glucoside)	C21H22O10	22.37	435.12912	
64	Flavonoid	Hyperoside (Quercetin-3-O-galactoside, Hyperin)	C21H20O12	22.65		463.08765
65	Miscellaneous	Naringenin chalcone isomer 2	C15H12O5	22.66	273.07630	
66	Flavonoid	Quercetin-3-O-glucuronide	C21H18O13	22.66		477.06692
67	Flavonoid	Isoquercitrin (Hirsutrin, Quercetin-3-O-glucoside)	C21H20O12	22.88		463.08765
68	Flavonoid	Laricitrin (Myricetin-3'-O-methyl ether)	C16H12O8	23.19	333.06105	
69	Flavonoid	Laricitrin-O-hexoside	C22H22O13	23.21		493.09822
70	Flavonoid	Avicularin (Quercetin-3-O-arabinofuranoside, Fenicularin)	C20H18O11	23.47		433.07709
71	Flavonoid	Myricetin	C15H10O8	24.18		317.02974
72	Flavonoid	Cyanidin-O-(coumaroyl)hexoside isomer 1	C30H26O13	24.31	595.14517	
73	Flavonoid	Quercitrin (Quercetin-3-O-rhamnoside)	C21H20O11	24.43	449.10839	
74	Flavonoid	Kaempferol-3-O-glucuronide	C21H18O12	24.59		461.07200
75	Flavonoid	Quercetin-O-rhamnoside-O-pentoside	C26H28O15	24.61	581.15065	
76	Flavonoid	Syringetin-O-hexoside	C23H24O13	24.98		507.11387

No.	Chemical classification	Compound	Molecular formula	RT	M [+]	M [-]
77	Flavonoid	Malvidin-O-(coumaroyl)hexoside isomer 1	C32H31O14	25.09	639.17139	
78	Flavonoid	Isorhamnetin-O-glucuronide	C22H20O13	25.13		491.08257
79	Terpenoid	Abscisic acid	C15H20O4	25.42		263.12834
80	Flavonoid	Cyanidin-O-(coumaroyl)hexoside isomer 2	C30H26O13	25.48	595.14517	
81	Miscellaneous	4-Methoxycinnamaldehyde	C10H10O2	25.74	163.07591	
82	Flavonoid	Malvidin-O-(coumaroyl)hexoside isomer 2	C32H31O14	26.13	639.17139	
83	Flavonoid	Peonidin-O-(coumaroyl)hexoside	C31H29O13	26.39	609.16082	
84	Flavonoid	Quercetin-O-(coumaroyl)hexoside	C30H26O14	26.82		609.12498
85	Flavonoid	Quercetin-3-O-[3-Hydroxy-3-methylglutaroyl-(→4)-rhamnoside]	C27H28O15	26.83		591.13500
86	Flavonoid	Pentahydroxyflavone	C15H10O7	27.02		301.03483
87	Flavonoid	Naringenin	C15H12O5	27.24		271.06065
88	Flavonoid	Trihydroxyflavanone	C15H12O5	28.28		271.06065

Table S4. Centralized list of fragmentation products for the compounds identified in the three ripe bilberry fruit extracts – aqueous (E1), methanolic (E2), and hydro-methanolic (E3).

**Putative identification**	**Fragm. 1**	**Fragm. 2**	**Fragm. 3**	**Fragm. 4**	**Fragm. 5**	**Fragm. 6**
2-Aminoadipic acid	144,0656	116,0709	98,0606	70,0658	55,0187	
4-Coumaric acid	119,0489	93,0331				
4-Methoxycinnamaldehyde	145,0649	135,0808	131,0492	121,0650	105,0704	55,0186
5-Hydroxymethyl-2-furaldehyde	109,0288	97,0290	81,0341	69,0342	53,0394	
5-Methyl-2-furaldehyde	93,0340	83,0497	81,0343	55,0550	53,0394	
7-Deoxyloganic acid	197,0816	153,0911	135,0804	113,0230	109,0652	89,0230
Abscisic acid	219,1385	204,1151	201,1279	152,0831	151,0752	139,0751
Adenine	119,0356	94,0404	92,0246	67,0299		
Ampelopsin (Ampeloptin, Dihydromyricetin)	193,0138	178,9979	153,0184	125,0232		
Arginine	158,0924	130,0977	116,0709	112,0872	70,0658	60,0564
Asparagine	116,0346	88,0399	87,0446	74,0243	70,0295	
Avicularin (Quercetin-3-O-arabinofuranoside, Fenicularin)	301,0358	300,0281	271,0252	255,0299	178,9974	151,0023
Caffeic acid	135,0440	107,0488				
Caffeoylshikimic acid	179,0342	161,0235	135,0441			
Catechin	245,0820	205,0505	203,0709	151,0390	125,0232	109,0282
Catechin or Epicatechin-O-hexoside	289,0725	245,0821	203,0708	151,0391	125,0233	109,0282
Chlorogenic acid (3-O-Caffeoylquinic acid)	163,0390	145,0284	135,0442	117,0337	107,0494	89,0389
Choline	60,0815	59,0736	58,0657			
Cinnamtannin B1	711,1375	693,1273	573,1052	559,0894	289,0725	125,0231
Cinnamtannin D1	711,1379	693,1243	573,1038	289,0721	245,0813	125,0232
Coumaroyl-shikimate	163,0390	155,0343	119,0488			
Coumaroylquinic acid	191,0556	173,0457	163,0388	119,0488	93,0332	
Cyanidin-3-O-arabinoside	287,0549	241,0507	213,0543			
Cyanidin-3-O-glucoside (Kuromanin, Asterin, Chrysanthemin)	287,0552	213,0546	137,0235			
Cyanidin-3-O-sambubioside (Sambicyanin, Gossypicyanin)	287,0550					
Cyanidin-O-(coumaroyl)hexoside	287,0552	213,0539	147,0438			
Delphinidin-3-O-arabinoside	303,0498	257,0440	229,0489			
Delphinidin-3-O-galactoside (Empetrin)	303,0502	257,0444	229,0493			
Delphinidin-O-(pentosyl)hexoside	303,0501	257,0443	229,0491			
Dihydroxy-methoxybenzoic acid	168,0055	140,0104				
Dimethoxy-hidroxycinnamic acid	207,0651	192,0418	179,0704	175,0389	147,0440	119,0493
Epicatechin	245,0819	205,0506	203,0712	151,0391	125,0231	109,0282
Epigallocatechin	261,0773	219,0660	179,0343	167,0340	137,0233	125,0231
Ferulic acid	178,0263	149,0598	137,0237	134,0363		
Feruloylquinic acid	193,0498	191,0556	173,0447	93,0331		
Gallic acid (3,4,5-Trihydroxybenzoic acid)	125,0231	97,0281	81,0332	79,0175	69,0331	

**Putative identification**	**Fragm. 1**	**Fragm. 2**	**Fragm. 3**	**Fragm. 4**	**Fragm. 5**	**Fragm. 6**
Gallocatechin	261,0773	219,0659	179,0342	167,0339	137,0232	125,0230
Glutamic acid	130,0501	102,0554	84,0450	56,0502		
Gulonic acid γ-lactone or δ-Gluconic acid δ-lactone	133,0497	125,0236	115,0393	97,0290	85,0290	73,0291
Histidine	110,0716	95,0609	93,0453	83,0610		
Hyperoside (Quercetin-3-O-galactoside, Hyperin)	301,0359	300,0279	271,0252	255,0298	178,9976	151,0025
Idaein (Idein, Cyanidin-3-O-galactoside)	287,0552	213,0542	137,0235			
Isoleucin or Leucin	86,0970	69,0706				
Isoquercitrin (Hirsutrin, Quercetin-3-O-glucoside)	301,0360	300,0281	271,0253	255,0302	178,9976	151,0025
Isorhamnetin-O-glucuronide	315,0519	300,0283	271,0254	243,0305		
Kaempferol-3-O-glucuronide	285,0409	229,0502	175,0236	113,0232		
Laricitrin (Myricetin-3'-O-methyl ether)	318,0376	301,0346	273,0396	245,0446	217,0497	153,0178
Laricitrin-O-hexoside	331,0465	330,0386	315,0152	287,0203	178,9976	
Lysine	130,0865	84,0813	67,0548			
Malvidin-3-O-arabinoside	331,0812	315,0500	299,0560	287,0547	270,0518	242,0567
Malvidin-O-(coumaroyl)hexoside	331,0812	315,0499	287,0548	270,0535	242,0575	
Malvidin-O-hexoside	331,0812	315,0499	299,0551	287,0550	270,0523	242,0573
Methyl gallate	168,0055	124,0153				
Myricetin	271,0259	178,9979	165,0184	151,0026	137,0234	109,0282
Myricetin-3-O-arabinoside	316,0231	287,0202	271,0252			
Myricetin-O-hexoside	317,0307	316,0229	287,0203	271,0251	242,0222	178,9978
Myricetin-O-pentoside isomer	316,0229	287,0206	271,0257			
N-(2-Phenylethyl)acetamide	122,0966	105,0703	103,0548	90,9481	79,0550	77,0391
Naringenin	177,0184	165,0174	151,0025	119,0490	107,0126	93,0332
Naringenin chalcone	171,0287	153,0183	147,0442	123,0444	119,0493	
Nicotinamide	106,0290	96,0448	80,0501			
Nicotinic acid (Niacin,B3)	96,0450	80,0501	78,0343			
Pantothenic acid (B5)	202,1076	184,0972	174,1121	116,0347	90,0555	72,0451
Pelargonidin-O-hexoside	271,0602					
Pentahydroxyflavone	273,0411	257,0456	229,0501	178,9979	151,0026	107,0125
Peonidin-3-O-arabinoside	301,0707	286,0472	258,0526			
Peonidin-O-(coumaroyl)hexoside	301,0709	286,0472	258,0524	147,0438		
Peonidin-O-(pentosyl)hexoside	301,0709	286,0474	258,0528			
Peonidin-O-hexoside	331,0811	301,0707	286,0472	258,0522	230,0576	201,0533
Peonidin-O-pentoside isomer	301,0709	286,0475	258,0527			
Petunidin-3-O-arabinoside	317,0656	302,0422	274,0473	245,0441	217,0495	203,0337
Petunidin-3-O-galactoside	317,0657	302,0422	274,0476	245,0446	217,0497	203,0335
Petunidin-O-(pentosyl)hexoside	317,0658	302,0428	287,0549	274,0473		
Phenylalanine	149,0597	131,0493	120,0810	107,0494	103,0546	93,0704
Phytosphingosine	300,2897	282,2794	270,2796	264,2667	252,2690	95,0858
Procyanidin B1 or B3	409,0922	287,0550	163,0390	139,0392	127,0392	123,0442

**Putative identification**	**Fragm. 1**	**Fragm. 2**	**Fragm. 3**	**Fragm. 4**	**Fragm. 5**	**Fragm. 6**
Prunin (Naringenin 7-O-glucoside)	273,0758	171,0287	153,0182	147,0441	119,0494	91,0548
Quercetin-3-O-[3-Hydroxy-3-methylglutaroyl-(→4)-rhamnoside]	489,1026	447,0935	301,0359	300,0279	271,0255	151,0027
Quercetin-3-O-glucuronide	301,0359	255,0299	178,9978	163,0027	151,0026	121,0281
Quercetin-O-(coumaroyl)hexoside	463,0885	301,0360	300,0281	271,0252	255,0301	151,0022
Quercetin-O-rhamnoside-O-pentoside	449,1077	413,0859	345,0610	303,0502	257,0451	
Quercitrin (Quercetin-3-O-rhamnoside)	303,0501	257,0443	229,0487	129,0549	85,0290	71,0498
Saccharic acid	191,0194	173,0081	147,0289	129,0183	111,0073	85,0280
Syringetin-O-hexoside	344,0541	329,0307	316,0591	301,0358	273,0408	242,0218
Threonine	102,0554	84,0450	74,0607	56,0503		
Trihydroxyflavanone	177,0190	165,0184	151,0026	119,0489	107,0125	93,0332
Tryptophan	188,0707	170,0601	159,0917	146,0600	132,0809	118,0653
Tyrosine	165,0548	147,0442	136,0759	123,0443	119,0495	91,0548
Vanillin	125,0600	111,0445	93,0340	65,0393		
γ-Aminobutyric acid	87,0446	86,0606	69,0341	58,0657		

Table S5. Phytochemicals found in extracts E1, E2, and E3, compared to substances previously reported in Vaccinium myrtillus or other Vaccinium spp.

**Putative identification**	**Vaccinium spp. and plant part**
Ampelopsin (Ampeloptin, Dihydromyricetin)	N/A
Avicularin (Quercetin-3-O-arabinofuranoside, Fenicularin)	*V. myrtillus* L. – fruit [35]
Catechin or Epicatechin-O-hexoside	*V. myrtillus* L. – fruit [35]
Cinnamtannin B1	*V. vitis-idaea* L. – fruit [36]
Cinnamtannin D1	*V. vitis-idaea* L. – fruit [36]
Cyanidin-3-O-arabinoside	*V. myrtillus* L. – fruit [37]
Cyanidin-3-O-glucoside (Kuromanin, Asterin, Chrysanthemin)	*V. myrtillus* L. – fruit [35]
Cyanidin-3-O-sambubioside (Sambicyanin, Gossypicyanin)	*V. myrtillus* L. – fruit [38]
Cyanidin-O-(coumaroyl)hexoside	N/A
Delphinidin-3-O-arabinoside	*V. corymbosum* – fruit<br>*V. myrtillus* L. – fruit [37]
Delphinidin-3-O-galactoside (Empetrin)	*V. corymbosum* – fruit [39]<br>*V. myrtillus* L. – fruit [37]
Delphinidin-O-(pentosyl)hexoside	N/A
Epicatechin	*V. myrtillus* L. – fruit [37]
Epigallocatechin	*V. myrtillus* L. – fruit [37]
Gallocatechin	*V. myrtillus* L. – stems [24]
Hyperoside (Quercetin-3-O-galactoside, Hyperin)	*V. corymbosum* – fruit [39]
Idaein (Idein, Cyanidin-3-O-galactoside)	*V. corymbosum* – fruit [39]<br>*V. myrtillus* L. – fruit [37]
Isoquercitrin (Hirsutrin, Quercetin-3-O-glucoside)	*V. myrtillus* L. – fruit [37]
Isorhamnetin-O-glucuronide	*V. myrtillus* L. – fruit [35]
Kaempferol-3-O-glucuronide	*V. myrtillus* L. – fruit [35]
Laricitrin (Myricetin-3'-O-methyl ether)	*V. myrtillus* L. – fruit [35]
Laricitrin-O-hexoside	*V. myrtillus* L. – fruit [40]
Malvidin-3-O-arabinoside	*V. corymbosum* – fruit [39]<br>*V. myrtillus* L. – fruit [35]
Malvidin-O-(coumaroyl)hexoside	N/A
Malvidin-O-hexoside	*V. corymbosum* – fruit [39]<br>*V. myrtillus* L. – fruit [37]
Myricetin	*V. myrtillus* L. – fruit [35]
Myricetin-3-O-arabinoside	*V. myrtillus* L. – fruit [40]
Myricetin-O-hexoside	*V. myrtillus* L. – fruit [35]
Myricetin-O-pentoside isomer	*V. myrtillus* L. – fruit [35]
Naringenin	N/A
Naringenin chalcone	N/A
Pentahydroxyflavone<br>(Hypolaetin, Quercetin, Tricetin)	*V. myrtillus* L. – fruit [35]
Peonidin-3-O-arabinoside	*V. myrtillus* L. – fruit [40]
Peonidin-O-(coumaroyl)hexoside	N/A
Peonidin-O-(pentosyl)hexoside	N/A
Peonidin-O-hexoside	*V. corymbosum* – fruit [39]<br>*V. myrtillus* L. – fruit [37]
Peonidin-O-pentoside isomer	N/A
Petunidin-3-O-arabinoside	*V. myrtillus* L. – fruit [37]<br>*V. myrtillus* L. – fruit [35]

**Putative identification**	**Vaccinium spp. and plant part**
Petunidin-3-O-galactoside	*V. corymbosum* – fruit [39]<br>*V. myrtillus* L. – fruit [37]<br>*V. myrtillus* L. – fruit [35]
Petunidin-O-(pentosyl)hexoside	N/A
Procyanidin B1 or B3	*V. myrtillus* L. – fruit [35]
Prunin (Naringenin 7-O-glucoside)	N/A
Quercetin-3-O-[3-Hydroxy-3-methylglutaroyl-(→4)-rhamnoside]	*V. myrtillus* L. – fruit [41]
Quercetin-3-O-glucuronide	*V. corymbosum* – fruit [39]<br>*V. myrtillus* L. – fruit [37]<br>*V. myrtillus* L. – fruit [35]
Quercetin-O-(coumaroyl)hexoside	N/A
Quercetin-O-rhamnoside-O-pentoside	*V. myrtillus* L. – fruit [35]
Quercitrin (Quercetin-3-O-rhamnoside)	*V. myrtillus* L. – fruit [35]
Syringetin-O-hexoside	*V. myrtillus* L. – fruit [35]
Trihydroxyflavanone	N/A
2-Aminoadipic acid	N/A
Arginine	N/A
Asparagine	N/A
Glutamic acid	N/A
Histidine	N/A
Isoleucine or Leucine	N/A
Lysine	N/A
Phenylalanine	N/A
Threonine	N/A
Tryptophan	N/A
Tyrosine	N/A
γ-Aminobutyric acid (GABA)	N/A
Caffeoylshikimic acid	*V. myrtillus* L. – fruit [41]
Chlorogenic acid (3-O-Caffeoylquinic acid)	*V. myrtillus* L. – fruit [37]<br>*V. myrtillus* L. – fruit [35]
Coumaroylquinic acid	*V. myrtillus* L. – fruit [35]
Coumaroyl-shikimate	*V. myrtillus* L. – fruit [35]
Feruloylquinic acid	*V. myrtillus* L. – fruit [35]
Gallic acid (3,4,5-Trihydroxybenzoic acid)	*V. myrtillus* L. – fruit [35]
4-Coumaric acid	*V. myrtillus* L. – fruit [35]
Caffeic acid	*V. myrtillus* L. – fruit [37]<br>*V. myrtillus* L. – fruit [35]
Dihydroxy-methoxybenzoic acid	N/A
Dimethoxy-hidroxycinnamic acid (Sinapic acid)	*V. myrtillus* L. – fruit [35]
Ferulic acid	*V. myrtillus* L. – fruit [35]
Adenine	N/A
Nicotinamide	*V. myrtillus* L. – fruit [35]
Nicotinic acid (B3)	N/A
Pantothenic acid (B5)	*V. myrtillus* L. – fruit [35]
Abscisic acid (ABA)	*V. myrtillus* L. – fruit [42]
Saccharic acid	N/A
Gulonic acid γ-lactone or δ-Gluconic acid δ-lactone	N/A
Methyl gallate	*V. myrtillus* L. – leaves [43]

**Putative identification**	**Vaccinium spp. and plant part**
Choline	N/A
7-Deoxyloganic acid	N/A
4-Methoxycinnamaldehyde	N/A
5-Hydroxymethyl-2-furaldehyde	*V. myrtillus* L. – fruit [44]
5-Methyl-2-furaldehyde	*V. myrtillus* L. – fruit [44]
N-(2-Phenylethyl)acetamide	N/A
Phytosphingosine	N/A
Vanillin	N/A

Note:

N/A = Non-applicable; no reference was found for the compound previously being identified in Vaccinium spp. in scientific literature.

Table S6. Summary of the analytical chemistry analysis of the extracts, with compounds pertaining to each extract (E1, E2, and E3) marked (× , ✓). Compounds are organized by chemical classification, along with the reported health implications and the model system used (i.e. biochemical and biophysical assays, cell culture, animal model, human trials).

Chemical classification	Putative identification	E1	E2	E3	Reported health implications
Flavonoids	Ampelopsin (Ampeloptin, Dihydromyricetin) [C]	×	×	✓	• ameliorates non-alcoholic streatohepatitis – human [45]<br>• antibacterial agent – bacteria, mouse, rat [46,47]<br>• anti-cancer agent – cell culture, mouse [48]<br>• anti-diabetic agent – rat [49]<br>• antioxidant – piglet [50]<br>• cardiovascular protective agent – mouse [51]<br>• hepatoprotective agent – mouse [52]<br>• immune response modulator – cell culture [53]<br>• neuroprotective agent – mouse [54]
	Avicularin (Quercetin-3-O-arabinofuranoside, Fenicularin) [A]	✓	✓	✓	• ameliorates obesity – cell culture [55]<br>• anti-allergic agent – human [56]<br>• anti-cancer agent – human [57]<br>• anti-diabetic agent – cell culture [55]<br>• antibacterial agent – bacteria [58]<br>• antidepressant – rat [59]<br>• antioxidant – cell culture [60]<br>• cardiovascular protective agent– cell culture [61]<br>• hepatoprotective agent – cell culture [61]<br>• immune response modulator – cell culture [62]
	Catechin or Epicatechin-O-hexoside [A]	✓	✓	✓	• anti-cancer agent – hamster, rat [63]<br>• anti-diabetic agent – rat [63]<br>• antioxidant – rat [63]<br>• cardiovascular protective agent – rat [63]<br>• iron absorption modulator – rat [63]
	Cinnamtannin B1 [B]	✓	✓	✓	• anti-cancer agent – cell culture [564]<br>• anti-diabetic agent – cell culture [65]<br>• antibacterial agent – bacteria [66]<br>• antioxidant – cell culture, biochemical and biophysical assays [67]<br>• antiviral agent – cell culture [66]
	Cinnamtannin D1 [B]	✓	✓	✓	• anti-cancer agent – cell culture [68]<br>• anti-diabetic agent – mouse, cell culture [69]<br>• antioxidant – biochemical and biophysical assays [70]<br>• immune response modulator – mouse, cell culture [71]
	Cyanidin-3-O-arabinoside [A]	✓	✓	✓	• antioxidant – cell culture [72]<br>• cardiovascular protective agent – cell culture and vascular rings [72]<br>• protective agent against epithelial dysfunction – cell culture [72]
	Cyanidin-3-O-glucoside (Kuromanin, Asterin, Chrysanthemin) [A]	✓	✓	✓	• ameliorates non-alcoholic streatohepatitis – mouse [73]<br>• anti-cancer agent – biochemical and biophysical assays [74], cell culture [75]<br>• anti-diabetic agent – cell culture [75]<br>• antioxidant – biochemical and biophysical assays [76]<br>• hepatoprotective agent – mouse [77]<br>• immune response modulator – cell culture [78]

Chemical classification	Putative identification	E1	E2	E3	Reported health implications
					• UV photoprotective agent – cell culture [79]
	Cyanidin-3-O-sambubioside (Sambicyanin, Gossypicyanin) [A]	✓	✓	✓	• antioxidant – cell culture [80]<br>• neuroprotective agent – cell culture [80]
	Cyanidin-O-(coumaroyl)hexoside [C]	×	✓	✓	N/A
	Delphinidin-3-O-arabinoside [A]	✓	✓	✓	• anti-diabetic agent – cell culture [81]<br>• epidermal growth factor receptor inhibitor – cell culture [82]
	Delphinidin-3-O-galactoside (Empetrin) [A]	✓	✓	✓	• epidermal growth factor receptor inhibitor – cell culture [82]<br>• hepatoprotective agent - mouse [83]
	Delphinidin-O-(pentosyl)hexoside [C]	×	✓	✓	N/A
	Epicatechin [A]	✓	✓	✓	• anti-cancer agent – cell culture [84]<br>• anti-diabetic agent – rat [85]<br>• antioxidant – biochemical and biophysical assays [86]<br>• cardiovascular protective agent – human [87, 88]<br>• improves endothelial function – human [89]
	Epigallocatechin [A]	✓	✓	✓	• anti-cancer agent – cell culture [90]<br>• antioxidant agent – biochemical and biophysical assays [86]<br>• cardiovascular protective agent – human [87]<br>• immune response modulator – cell culture [91]<br>• UV photoprotective agent – human [92]
	Gallocatechin [A]	✓	✓	✓	• anti-cancer agent – cell culture [90]<br>• antioxidant – biochemical and biophysical assays [86]<br>• cardiovascular protective agent – human [87]<br>• immune response modulator – cell culture [91]
	Hyperoside (Quercetin-3-O-galactoside, Hyperin) [B]	✓	✓	✓	• anti-cancer agent – cell culture [93]<br>• anti-diabetic agent – rat [94]<br>• anti-nociceptive agent – biochemical and biophysical assays, cell culture, mouse [95]<br>• antidepressant – cell culture [96]<br>• antioxidant – cell culture [97]<br>• antiviral agent – cell culture [98]<br>• cardiovascular protective agent – rat [99]<br>• cytochrome P450 inhibitor – biochemical and biophysical assays, cell culture [100]<br>• gastroprotective agent – mouse [101]<br>• hepatoprotective agent – mouse [102]<br>• immune response modulator – mouse [103, 104]<br>• neuroprotective agent – cell culture, rat [105,106]
	Idaein (Idein, Cyanidin-3-O-galactoside) [A]	✓	✓	✓	• ameliorates obesity – mouse [107]<br>• anti-cancer agent – cell culture [108]<br>• antioxidant – biochemical and biophysical assays, cell culture [109], rat [110]<br>• neuroprotective agent – mouse [111]
	Isoquercitrin (Hirsutrin, Quercetin-3-O-glucoside) [A]	✓	✓	✓	• anti-allergic agent – guinea pig [112]<br>• anti-asthmatic agent – guinea pig [112]

Chemical classification	Putative identification	E1	E2	E3	Reported health implications
					• anti-cancer agent – mouse [113]<br>• anti-diabetic agent – rat [114]<br>• antioxidant – mouse [115]<br>• cardiovascular protective agent – rat [116]<br>• immune response modulator – rat [117]<br>• sedative – mouse [118]
	Isorhamnetin-O-glucuronide [A]	✓	✓	✓	• antioxidant – cell culture [119]<br>• cardiovascular protective agent – rat [120]<br>• immune response modulator – cell culture [119]
	Kaempferol-3-O-glucuronide [A]	✓	✓	✓	• ameliorates obesity – guinea pig [121]<br>• angiotensin converting enzyme inhibitor – cell culture [122]<br>• anti-cancer agent – cell culture [123]<br>• anti-diabetic agent – rat [124]<br>• antibacterial agent – bacteria, gerbil [125]<br>• antioxidant – C.elegans [126], cell culture [127]<br>• cardiovascular protective agent – cell culture [127], rat [128]<br>• immune response modulator – cell culture [129]<br>• increases thermotolerance – C. elegans [126]
	Laricitrin (Myricetin-3'-O-methyl ether) [A]	✓	✓	✓	• anti-cancer agent – cell culture [130]<br>• immune response modulator – cell culture [130]
	Laricitrin-O-hexoside [A]	✓	✓	✓	N/A
	Malvidin-3-O-arabinoside [A]	✓	✓	✓	N/A
	Malvidin-O-(coumaroyl)hexoside [C]	×	✓	✓	N/A
	Malvidin-O-hexoside [A]	✓	✓	✓	N/A
	Myricetin [A]	×	✓	✓	• anti-cancer agent – mouse [131]<br>• anti-diabetic agent – cell culture, rat [132, 133]<br>• antioxidant – biochemical and biophysical assays, cell culture [134]<br>• antiviral agent – biochemical and biophysical assays, cell culture [135]<br>• mutagenic agent – cell culture [136]
	Myricetin-3-O-arabinoside [A]	✓	✓	✓	N/A
	Myricetin-O-hexoside	✓	✓	✓	N/A
	Myricetin-O-pentoside isomer [A]	✓	✓	✓	N/A
	Naringenin [C]	✓	✓	✓	• anti-cancer agent – cell culture, rat [137, 138]<br>• anti-diabetic agent – mouse [139]<br>• antidepressant – mouse [140]<br>• antifilarial agent – worms, microfilaria [141]<br>• antioxidant – rat [142]<br>• cardiovascular protective agent – rat [143]<br>• immune response modulator – cell culture [144]
	Naringenin chalcone [C]	×	✓	✓	• anti-allergic agent – mouse [145]<br>• anti-cancer agent – cell culture [146]<br>• antioxidant – biochemical and biophysical assays [147]<br>• immune response modulator – cell culture [148]

Chemical classification	Putative identification	E1	E2	E3	Reported health implications
	Pentahydroxyflavone (Hypolaetin, Quercetin, Tricetin) [A]	✓	✓	✓	• anti-cancer agent – rat [149]<br>• antioxidant – mouse [150]<br>• cardiovascular protective agent – cell culture [151], rat [152]<br>• immune response modulator – human [153]
	Peonidin-3-O-arabinoside [A]	✓	✓	✓	N/A
	Peonidin-O-(coumaroyl)hexoside	×	✓	✓	N/A
	Peonidin-O-(pentosyl)hexoside [C]	×	✓	×	N/A
	Peonidin-O-hexoside [A]	✓	✓	✓	N/A
	Peonidin-O-pentoside isomer [C]	×	✓	×	N/A
	Petunidin-3-O-arabinoside [A]	✓	✓	✓	N/A
	Petunidin-3-O-galactoside [A]	✓	✓	✓	N/A
	Petunidin-O-(pentosyl)hexoside [C]	×	✓	×	N/A
	Procyanidin B1 or B3 [A]	✓	✓	✓	• antioxidant – biochemical and biophysical assays, cell culture [154]<br>• immune response modulator – cell culture [119]
	Prunin (Naringenin 7-O-glucoside) [C]	✓	✓	✓	• anti-diabetic agent – cell culture, mouse [155, 156]<br>• antioxidant – cell culture [157]<br>• cardiovascular protective agent – cell culture [158]
	Quercetin-3-O-[3-Hydroxy-3-methylglutaroyl-(→4)-rhamnoside] [A]	✓	✓	✓	N/A
	Quercetin-3-O-glucuronide [A]	✓	✓	✓	• angiogenesis inhibitor - biochemical and biophysical assays, cell culture [159]<br>• anti-diabetic agent – biochemical and biophysical assays, cell culture [160]<br>• antioxidant – rat [161]<br>• immune response modulator – biochemical and biophysical assays, cell culture [160]<br>• neuroprotective agent – mouse [162]
	Quercetin-O-(coumaroyl)hexoside [C]	×	✓	✓	N/A
	Quercetin-O-rhamnoside-O-pentoside [A]	✓	✓	✓	N/A
	Quercitrin (Quercetin-3-O-rhamnoside) [A]	✓	✓	✓	• antibacterial agent – bacteria 5 [163]<br>• antioxidant – biochemical and biophysical assays [164]<br>• UV photoprotective agent – biochemical and biophysical assays [165]
	Syringetin-O-hexoside [A]	✓	✓	✓	N/A
	Trihydroxyflavanone [C]	×	✓	✓	• anti-cancer agent – cell culture, rat [127, 138]<br>• anti-diabetic agent – mouse [139]<br>• antidepressant – mouse [140]<br>• antifilarial agent – worms, microfilaria [141]<br>• antioxidant – rat [142]

**Chemical classification**	**Putative identification**	**E1**	**E2**	**E3**	**Reported health implications**
					• cardiovascular protective agent – rat [143]<br>• immune response modulator – cell culture [144]
Amino acids	2-Aminoadipic acid [C]	×	✓	✓	degradation product of lysine<br>• anti-diabetic agent – mouse [164]<br>• marker for aging – human [165]<br>• marker for increased risk of developing diabetes – human [166]<br>• marker for sepsis – human [167]<br>• oxidative stress inducer – rat [168]
	Arginine [C]	✓	✓	✓	conditionally essential amino acid in adults, creatine nitric oxide and ornithine precursor<br>• anti-diabetic agent – rat [169], rabbit [170]<br>• cardiovascular protective agent – mouse, rabbit [171]<br>• immune response modulator – mouse [172]
	Asparagine [C]	×	✓	✓	non-essential amino acid, precursor of ammonia, potential glycosylation site in the ER<br>• metastasis promoter – mouse [173]<br>• neuroprotective agent – human [174]
	Glutamic acid [C]	✓	×	✓	non-essential amino acid; responsible for the umami taste; neurotransmitter; precursor of GABA<br>• ammonia level modulator – biochemical and biophysical assays [175]<br>• osmotic anionic balance modulator – rat [176]
	Histidine [C]	✓	×	✓	essential amino acid, found in abundance in the active site of proteins; precursor of histamine<br>• food intake suppressor – rat [177]<br>• marker for rheumatoid arthritis – human [178]
	Isoleucin or Leucin [C]	×	✓	✓	essential amino acid; precursor of alanine and glutamine<br>• ameliorates obesity – human [179]<br>• ameliorates sarcopenia – rat [172], human [181]<br>• anti-diabetic agent – human [182]
	Lysine [C]	✓	×	✓	essential amino acid; glutamate precursor<br>• neuroprotective agent – mouse [183]
	Phenylalanine	✓	✓	✓	essential amino acid; tyrosine, phenethylamine, adrenaline, noradrenaline, melanin precursor<br>• marker for phenylketonuria – human [184]<br>• neurotoxic agent – human [185]
	Threonine [C]	✓	✓	✓	essential amino-acid; precursor of glycine; prodrug of mefenamic acid<br>• analgesic – rat [186]<br>• immune response modulator – rat [186]
	Tryptophan [C]	✓	✓	✓	essential amino-acid, precursor of serotonin and melatonin<br>• antidepressant – human [187]<br>• anxiolytic – human [187]<br>• potential sleep aid – human [187]
	Tyrosine [C]	✓	✓	✓	non-essential amino acid, found mainly in proteins involved in signal transduction pathways, as the residue can be phosphorylated/sulfated; precursor of L-DOPA, melanin, coenzyme Q10

**Chemical classification**	**Putative identification**	**E1**	**E2**	**E3**	**Reported health implications**
					• ameliorates symptoms under stress (e.g. sleep deprivation, multitasking, coldness, fatigue, weight loss) – human, mouse [188]
	γ-Aminobutyric acid (GABA) [C]	✓	×	×	• anti-diabetic agent – intact human pancreatic islets [189]<br>• immune response modulator – cell culture [190]<br>• neuroprotective agent – mouse [191]
Polyphenols	Caffeoylshikimic acid [A]	✓	✓	✓	N/A
	Chlorogenic acid (3-O-Caffeoylquinic acid) [A]	✓	✓	✓	• ameliorates dyslipidemia – rat [192]<br>• ameliorates obesity – mouse [193]<br>• anti-cancer – cell culture [194]<br>• anti-diabetic agent – human, mouse [195, 196]<br>• antibacterial agent – biochemical and biophysical assays [195]<br>• antioxidant – biochemical and biophysical assays [195]<br>• cardiovascular protective agent – mouse [195]; human [197]<br>• genotoxic agent – biochemical and biophysical assays, bacteria [198]<br>• neuroprotective agent – mouse [195]
	Coumaroylquinic acid [A]	✓	✓	✓	N/A
	Coumaroyl-shikimate [A]	✓	✓	✓	N/A
	Feruloylquinic acid [A]	✓	✓	✓	N/A
	Gallic acid (3,4,5-Trihydroxybenzoic acid) [A]	✓	✓	✓	• anti-cancer agent – cell culture, mouse [199], biochemical and biophysical assays [200]<br>• antioxidant – biochemical and biophysical assays [201]<br>• immune response modulator – mouse [202]<br>• neuroprotective agent – cell culture, biochemical and biophysical assays [203]
Carboxylic acids	4-Coumaric acid [A]	✓	✓	✓	• anti-cancer agent – rabbit [204]<br>• antioxidant – rabbit [204]<br>• immune response modulator – rat [205]<br>• male contraceptive – mouse [206]<br>• neuroprotective agent – rat [207]
	Caffeic acid [A]	✓	✓	✓	• antioxidant – biochemical and biophysical assays [208]<br>• immune response modulator – cell culture [209]
	Dihydroxy-methoxybenzoic acid [C]	×	✓	✓	N/A
	Dimethoxy-hidroxycinnamic acid (Sinapic acid) [A]	✓	✓	✓	• antioxidant – cell culture [210]<br>• immune response modulator – mouse [211]
	Ferulic acid [A]	✓	✓	✓	• antioxidant – biochemical and biophysical assays [212]<br>• immune response modulator – cell culture [212]<br>• UV photoprotective agent - human [212]
Vitamins	Adenine [C]	×	✓	✓	• anti-diabetic agent – rabbit [213]<br>• antiviral agent – cell culture, bacteria [214]<br>• cardiovascular protective agent – human, cat, rat [213]<br>• diuretic agent – dogs [213]<br>• increases muscle creatine levels – mouse [213]<br>• treats agranulocytosis and leukopenia – human [213]

**Chemical classification**	**Putative identification**	**E1**	**E2**	**E3**	**Reported health implications**
	Nicotinamide [A]	✓	✓	✓	• ameliorates alcoholic liver disease – mouse [215]<br>• ameliorates granuloma annulare – human [216]<br>• ameliorates necrobiosis lipoidica – human [217]<br>• ameliorates pemphigoid – human [218]<br>• ameliorates polymorphous light eruption – human [219]<br>• anti-diabetic agent – human [220]<br>• neuroprotective agent – human [221]
	Nicotinic acid (B3) [C]	×	✓	✓	• ameliorates erectile dysfunction – human [222]<br>• anti-diabetic agent – rat [223]<br>• cardiovascular protective agent– human [224]<br>• neuroprotective agent – human [225]<br>• prevents congenital malformations – mouse [226]<br>• ameliorates dyslipidemia – human [224]<br>• treats pellagra – human [227]
	Pantothenic acid (B5) [A]	×	×	✓	• ameliorates multiple sclerosis – mouse [228]<br>• ameliorates muscular cramps in pregnant women – human [229]<br>• ameliorates obesity – rats [230]<br>• anti-diabetic agent – mouse [231, 232]<br>• cardiovascular protective agent – human [233]<br>• immune response modulator – human [234]<br>• maintenance treatment for atopic dermatitis – human [235]
Terpenoids	Abscisic acid (ABA) [A]	✓	✓	✓	• anti-diabetic agent – rat [236]<br>• neuroprotective agent – rat [237]<br>• ameliorates attention deficit disorder – rat [238] ameliorates prostate enlargement – rat [238]<br>• immune response modulator – rat [238]
Sugars	Saccharic acid [C]	✓	✓	✓	N/A
Lactones	Gulonic acid γ-lactone or δ-Gluconic acid δ-lactone [C]	✓	✓	✓	N/A
Esters	Methyl gallate [A]	✓	✓	✓	• anti-cancer agent – cell culture [239]<br>• antioxidant – cell culture [240]<br>• immune response modulator – mouse [241]
Alkaloids	Choline [C]	✓	×	×	• choline-phospholipids various functions – human [242]<br>• control of neural and muscle function as acetylcholine – human [243]<br>• ameliorates non-alcoholic streatohepatitis – mouse, rat, cell culture [244]<br>• lipotropic agent – rats [245]
Iridoids	7-Deoxyloganic acid [C]	×	✓	✓	• immune response modulator – biochemical and biophysical assays [246]
Miscellaneous	4-Methoxycinnamaldehyde [C]	×	×	✓	• anti-cancer agent – cell culture [247]<br>• cardiovascular protective agent – rat and cell culture [248]<br>• immune response modulator – cell culture [249]
	5-Hydroxymethyl-2-furaldehyde [A]	✓	✓	✓	immediate and complete elimination from the body – rat [250]

Chemical classification	Putative identification	E1	E2	E3	Reported health implications
	5-Methyl-2-furaldehyde [A]	✓	✓	✓	N/A
	N-(2-Phenylethyl)acetamide [C]	✓	×	×	N/A
	Phytosphingosine [C]	×	✓	×	• anti-cancer agent – cell culture [251]<br>• anti-melanogenic agent – cell culture [252]<br>• immune response modulator – mouse [253]
	Vanillin [C]	×	✓	✓	• neuroprotective agent – mouse [254]<br>• anti-diabetic agent – biochemical and biophysical assays and cell culture [255]<br>• ameliorates sickle cell anemia – biochemical and biophysical assays, cell culture [256, 257]<br>• anti-cancer agent – mouse, cell culture, and biochemical and biophysical assays [258]<br>• antioxidant – biochemical and biophysical assays [259], rat [260]<br>• immune response modulator – rat [260]<br>• hepatoprotective agent – rat [260]<br>• ameliorates obesity – rat [261]

Note:

N/A = Non-applicable; no reference was found for the compound to have a biological activity according to scientific literature.

*A The phytochemical was previously reported in Vaccinium myrtillus (**Table S5**)*

*B The phytochemical was previously reported in other Vaccinium spp., but not in Vaccinium myrtillus (**Table S5**)*

*C The phytochemical was not previously reported in any Vaccinium spp. (**Table S5**)*

Table S7. PCR primer sequences for the genes used in the experiments, alongside their efficiency.

Oligo name	Sequence	Efficiency	Probe
ilp5_qPCR_F1	ATGGACATGCTGAGGGTTG	1,53	123
ilp5_qPCR_R1	AATCGAATAGGCCCAAGGTG		123
ilp6_qPCR_F1	GGCCCTTGGCGATGTATTTC	1,8	4
ilp6_qPCR_R1	AATCGGTTACGTTCTGCAAGTC		4
ilp2_qPCR_F1	CCGACAGCGATCTGGAC	1,68	63
ilp2_qPCR_R1	CACTTCGCAGCGGTTCC		63
SIFa_qPCR_F1	ACTCACTCTGCTCCTGGTC	2	46
SIFa_qPCR_R1	AGTCTAGGCTGTTGCGTTTG		46
pdf_qPCR_F1	AACGATGCGGGCAAGTAAG	1,7	161
pdf_qPCR_R1	CAGGTTCCATCTTTCAGTGGTG		161
CNMa_qPCR_F1	CTACGGCGACCAGTTGAAG	2	18
CNMa_qPCR_R1	ACTTCCTGGCGAAAGCC		18
Thor F	CCAGATGCCCGAGGTGTA	1.8	22
Thor R	AGCCCGCTCGTAGATAAGTTT		22
tobi F	TGGATTCTGGTGAACCTGTG	2	138
tobi R	TCGCCCAAAAGGAACTCAT		138
InR F	ACGACAACAAAACCGTTGC	2.00	141
InR R	TTCACGTGATCTCAATCATGC		141
Akh_qPCR_F1	ATTGCAGCCGTGCTCTTC	1.82	47
Akh_qPCR_R1	AGGTTCCAGGACCAGCTC		47
Nplp3_qPCR_F1	TGTTCAAGCTGTGCGTCTTC	1.84	24
Nplp3_qPCR_R1	AATGAGACCAGGAGCAGGAG		24
Eip75B F	ATTACGGCGTGCATTCCT	1.68	58
Eip75B R	GGGCGATACTGGATCTTTTG		58
Hr96 F	CCAGCGAGGCTCTTTATGAT	1.78	153
Hr96 R	CGAGTGTCGTCGGGCTTA		153
Ide F	AGAACCTAATTCTTCGACTGCTG	1.67	99
Ide R	GTCCGCGGAGATTTATGAGT		99
CCAP_qPCR_F1	ATCATGTCGCAGGCCAAA	2.00	147
CCAP_qPCR_R1	TTTGCCGAAAGATTTCCTTGC		147
Npc2g_qPCR_F1	TTCGACTTCACGCCGAAC	2.00	117
Npc2g_qPCR_R1	CTCCACATTCTCGCTCTTGG		117
CCHa2_qPCR_F1	CCATATCTTTGCTACTGGTCGTTATC	1.85	162
CCHa2_qPCR_R1	ACCGTAGGCCTGGCATC		162
AstC_qPCR_F1	CCAGGCAATCTATCCCAATATTCC	2.00	140
AtsC_qPCR_R1	CTCCTCAAATAGGCGCTGTAAC		140
AstA F	TTCAGCGGCATTTGGAAT	1.90	66
AstA R	GAAGGGAGTTCATTGTGATGC		66
CG15618 F	CTTCCTGGCGGATCTCCT	2.00	164
CG15618 R	CGCATTGTCCGAGAAGAGTT		164
Irk1 F	AACGCTTCGAAGTGGTGGT	1.80	67
Irk1 R	CTGTGTGGTCATGCCAGTG		67
Usp_qPCR__F1	AATAAACAACCGTCACCTTGC	1.80	71
Usp_qPCR_R1	TTAAAGCGCTAATGCCCATC		71

Table S8. Hemolymph trehalose levels for *Drosophila melanogaster* Canton Special exposed to high sugar diet with and without supplementation of hydro-methanolic bilberry extract (E3). Data reported as average of triplicate experiments.

**Media used**	**Mean hemolymph trehalose level [mg/L]**	**Standard deviation [mg/L]**
Normal diet + hydro-methanolic bilberry extract (E3)	289	6.16
High sugar diet + hydro-methanolic bilberry extract (E3)	447	35.12
Normal diet control	457	14.26
High sugar diet control	943	34.98

Table S9. Preference index for hydro-methanolic bilberry extract (E3) supplementation for *Drosophila melanogaster* Canton Special compared to normal media alone. Data reported as average of triplicate experiments.

**Media used**	**Mean preference index**	**Standard deviation**
Normal diet + hydroxy-methanolic bilberry extract (E3) - *red*	0.43	0.02
Normal diet control - *blue*	0.48	0.04
Normal diet + hydroxy-methanolic bilberry extract (E3) - *blue*	0.52	0.04
Normal diet control - *red*	0.47	0.02
